# Platelet-derived extracellular vesicles aggravate septic acute kidney injury via delivering ARF6

**DOI:** 10.7150/ijbs.87165

**Published:** 2023-10-02

**Authors:** Xun Lu, Guiya Jiang, Yue Gao, Qi Chen, Si Sun, Weipu Mao, Nieke Zhang, Zepeng Zhu, Dong Wang, Guangyuan Zhang, Ming Chen, Lei Zhang, Shuqiu Chen

**Affiliations:** 1 Department of Urology, Affiliated Zhongda hospital of Southeast University, Nanjing, China.; 2 Surgical Research Center, Institute of Urology, School of Medicine, Southeast University, Nanjing, China.; 3 Department of Interventional Radiology and Vascular Surgery, Affiliated Zhongda hospital of Southeast University, Nanjing, China.

**Keywords:** sepsis, extracellular vesicles, platelet, acute kidney injury, ARF6

## Abstract

Circulating plasma extracellular vesicles (EVs) mostly originate from platelets and may promote organ dysfunction in sepsis. However, the role of platelet-derived EVs in sepsis-induced acute kidney injury (AKI) remains poorly understood. The present study extracted EVs from the supernatant of human platelets treated with phosphate buffer saline (PBS) or lipopolysaccharide (LPS). Then, we subjected PBS-EVs or LPS-EVs to cecal ligation and puncture (CLP) mice* in vivo* or LPS-stimulated renal tubular epithelial cells (RTECs)* in vitro*. Our results indicated that LPS-EVs aggravate septic AKI via promoting apoptosis, inflammation and oxidative stress. Further, ADP-ribosylation factor 6 (ARF6) was identified as a differential protein between PBS-EVs and LPS-EVs by quantitative proteomics analysis. Mechanistically, ARF6 activated ERK/Smad3/p53 signaling to exacerbate sepsis-induced AKI. LPS upregulated ARF6 in RTECs was dependent on TLR4/MyD88 pathway. Both genetically and pharmacologically inhibition of ARF6 attenuated septic AKI. Moreover, platelets were activated by TLR4 and its downstream mediator IKK controlled platelet secretion during sepsis. Inhibition of platelet secretion alleviated septic AKI. Collectively, our study demonstrated that platelet-derived EVs may be a therapeutic target in septic AKI.

## Introduction

Sepsis is a life-threatening organ dysfunction characterized by a dysregulated response to infection 1,2. Acute kidney injury (AKI) with an incidence of 40-50% is one of the most frequent complications among septic patients. In critically ill patients, sepsis-induced AKI is associated with a significantly high mortality and could contribute to the development of chronic kidney disease, which is a serious threat to human health 3-5. Although many efforts have been devoted to prevent the incidence of septic AKI, there is currently no effective and targeted therapy in the face of complex pathophysiology of septic AKI 6.

Previous studies have indicated that inflammation and apoptosis in kidneys are hallmarks of septic AKI 7,8. During sepsis, pattern recognition receptors (PRPs) in renal tubular epithelial cells (RTECs) were able to recognize damage associated molecular patterns (DAMPs) and pathogen associated molecular patterns (PAMPs) to promote the downstream expression of proinflammatory cytokines, subsequently initiating cell death in RTECs 9. Until now, the standard models of sepsis included cecal ligation and puncture (CLP) and lipopolysaccharide (LPS) injection 10-12. Toll-like receptor 4 (TLR4) and its downstream mediator myeloid differentiation primary response gene 88 (MyD88) is reported to be activated by LPS, mediating inflammation and apoptosis during septic AKI 13,14. Moreover, as a downstream effector of TLR4/MyD88 pathway, mitogen-activated protein kinases (MAPKs) also play important roles in kidney diseases 15-17.

Platelets are shed during the maturation of megakaryocyte, which not only maintain homeostasis, but also participate in inflammation either through direct contact or by releasing soluble mediators 18,19. Platelet particles are extracellular vesicles (EVs) released from activated platelets and the size of diameter varies between 100 and 1000 nm 20-22. Platelet-derived EVs, which account for about 70% of the total plasma EVs in patients with sepsis, are the main functional component of plasma EVs and have been reported to regulate intercellular communication during sepsis 23-26.

Previous studies have suggested a critical role of *N*-ethylmaleimide sensitive factor attachment protein receptors (SNAREs) complex formation in regulating platelet secretion and EVs release 27. Synaptosomal-associated protein 23 (SNAP 23) and syntaxin 2 (STX-2) are the core components in SNAREs complex, which are required for membrane fusion and subsequent granule release 28-30. However, whether platelet-derived EVs contribute to septic AKI requires further elucidation.

In present study, we aimed to explore the role of platelet-derived EVs during sepsis-induced AKI. Our results showed that LPS activated platelets via TLR4 and subsequently IκB controlled platelet secretion. ARF6 was enriched in LPS-EVs, which further promoted inflammation, apoptosis, and oxidative stress via phosphorylating of ERK, Smad3, and p53 to aggravate septic AKI.

## Materials and Methods

### Extracellular vesicle isolation

Human blood samples were from healthy volunteers. The platelets were extracted in EDTA-anticoagulated venous blood in a differential centrifugation method according to previously described 31. The detailed characteristics of included subjects was shown in Table S1. The isolated platelets at 1×10^8^ cells/ml were resuspended in modified Tyrode buffer (Sigma, USA) and treated with PBS or LPS (1 μg/ml) at 37°C for 3 hours (h). The supernatant of platelets was harvested and subjected to ultracentrifugation (Beckman Coulter, Fullerton, USA) at 20,000×g for 90 minutes (min) at 4°C to precipitate EVs. Finally, the pellets containing platelet-derived EVs were dissolved in 100 ml filtered PBS. The study was approved by the institutional ethics and review board of Zhongda Hospital, and informed consent was obtained from the healthy volunteers (2022ZDSYLL439-P01).

### Animals

Wild-type (WT) C57BL/6J male mice aged 6-8 weeks were obtained from Vital River Laboratories (Nanjing, China). CLP was conducted to induce polymicrobial sepsis according to previous study 10. The mice kidneys and blood samples were collected after 24 h of CLP. The mice were randomly divided into the following groups: Sham group, CLP group, CLP+PBS-EVs group and CLP+LPS-EVs group (n = 4-6 in each group). EVs were injected to mice via tail vein. All the experiments and procedures were approved by the Committee on the Ethics of Animal Experiments of Southeast University (Nanjing, China).

### Chemicals and reagents

TAK242, BMS-345541, BAY 11-7082, and SecinH3 were acquired from MedChemExpress (Shanghai, China). LPS (L2880) from Escherichia coli 055:B5 was obtained from Sigma (St. Louis, USA). Recombinant human ARF6 was purchased from Proteintech (Wuhan, China).

### Renal function analysis

For serum collection, blood samples were left at room temperature for 2 h and then centrifuged at 4°C of 3000 rpm for 15 min. Serum creatinine (SCr) and blood urea nitrogen (BUN) levels were measured based on a commercial assay kit (Jiancheng, Nanjing, China).

### Histologic examination

Mice kidneys were fixed in 4% paraformaldehyde (Servicebio, Wuhan, China) and sectioned into 4-μm thickness after embedment in paraffin. Tubular injury was evaluated after Periodic acid-Schiff (PAS) staining and measured in a double blinded manner. The severity of tubular damage was scored as 0, 1, 2, 3, and 4, indicating the percentage of injured renal tubules of 0%, 25%, 50%, 51-75%, >75%, respectively 8.

### Immunohistochemistry

Mice kidney samples were incubated with H_2_O_2_ before treatment with anti-NGAL antibody (1:100, Abcam, USA), anti-MPO antibody (1:500, Servicebio, China) anti-Ly6G antibody (1:500, Servicebio, China) and anti-F4/80 antibody (1:500, Servicebio, China). The slices were washed by PBS before incubating with a secondary antibody. The positive areas of NGAL and positive cells of MPO, Ly6G and F4/80 were measured under a light microscope and calculated by ImageJ software (version 1.51, USA).

### TUNEL staining

The embedded kidney samples were subjected to terminal deoxynucleotidyl transferasemediated dUTP nick-end labeling (TUNEL) assay (Millipore, USA) to detect the apoptic cells in the kidneys, according to the instructions. TUNEL positive cells in kidneys were calculated from 5 random images of each group.

### Quantitative real-time PCR analysis

TRIzol (Vazyme, Nanjing, China) was used to extract total RNA in the study. SweScript RT II First Strand cDNA Synthesis Kit (Servicebio, Wuhan, China) was used to synthetic cDNA according to manual. SYBR Green qPCR Master Mix (Servicebio, Wuhan, China) was added in conducting quantitative real-time PCR analysis. The utilized primers for detecting genes in the study were shown in Table S2. To calculate relative mRNA expression, 2^-ΔΔCt^ method was used and the internal reference gene was GAPDH.

### Western blot analysis

Radioimmunoprecipitation assay (RIPA) buffer (Servicebio, Wuhan, China) was used to extract proteins. Protein concentration was quantified by a BCA protein assay kit (Keygen Biotech, Nanjing, China). Western blot analysis was conducted according to our previous report 32, and the densitometry of proteins was calculated by ImageJ software. The primary antibodies used in this part were listed in Table S3.

### Transmission electron microscopy (TEM)

The EVs were placed onto a copper electron microscopy grid, which had been washed three times with filtered PBS before observation. The morphology of platelet-derived EVs was captured by TEM (HITACHI H7650 TEM; Tokyo, Japan) at ×40,000 magnification. In order to detect renal mitochondrial morphology, mice kidneys were fixed in 2.5% glutaraldehyde (Servicebio, Wuhan, China) immediately and subsequently treated according to standard procedures. ImageJ software was used to measure mitochondrial length to width ratio and average area according to a previous study 33.

### Nanoparticle tracking analysis (NTA)

Purified EVs from supernatant of platelets were sent to ZetaView program (Particle Metrix, Germany) for determining size distributions and particle concentrations.

### Bioluminescence imaging

LPS-EVs and PBS-EVs were stained with PKH26 (Sigma, USA). Tail vein injection of 100 μg PKH26-labeled EVs to mice was conducted in the study. The excised organs including heart, liver, spleen, lung and kidney were rinsed with PBS and imaged by using IVIS imaging system (Xenogen, USA).

### Cell culture and treatments

Human kidney tubular epithelium cells (HK-2) and mouse kidney tubular epithelium cells (TCMK-1) purchased from Shanghai Institutes for Biological Sciences (Shanghai, China) were maintained in Dulbecco's Modified Eagle Medium/Nutrient Mixture F-12 (DMEM/F12) medium (Keygen Biotech, Nanjing, China), supplementing with 10% foetal bovine serum (FBS, ExCell Bio, Shanghai, China), 1% streptomycin and penicillin antibiotics (Keygen Biotech, Nanjing, China) at 37℃ of 5% CO_2_ in a humidified atmosphere. For EVs co-culture, HK-2 and TCMK-1 cells were replaced with conditioned media supplemented with exosome-depleted FBS (System Biosciences, Palo Alto, CA, USA).

### Confocal microscopy

For EVs uptake experiments, purified EVs labeled with PKH26 were incubated with HK-2 cells for indicated times. Images were taken by a Zeiss LSM 700 confocal microscope.

### Cell transfection

HK-2 cells were transfected with small interfering RNA (siRNA) by transfection reagent (jetPRIME-Transfection reagent, New York, USA), accordingly. The sequences of siRNAs (GenePharma, Shanghai, China) used in the study were listed in Table S4.

### Cell viability analysis

Cell viability was determined by the Cell Counting Kit-8 assay (CCK-8, Biosharp, China) according to the manufacturer's instructions. HK-2 and TCMK-1 cells were seed into a 96-well plate at a density of 5000 cells/well. After treating with LPS and EVs, the medium was added with 10 μl CCK-8 solution and incubated for 2 h. Finally, the cell viability was measured at the absorbance of 450 nm.

### Flow cytometry analysis

To detect the proportion of platelets, the EVs were stained with the platelet-specific antibody of APC anti-CD61 Antibody (BioLegend, San Diego, CA, USA). The apoptosis rate of cultured HK-2 and TCMK-1 cells was detected by staining with an Annexin V/PI kit (Vazyme, Nanjing, China). For determining the production of reactive oxygen species (ROS), HK-2 cells were incubated with 2',7' -Dichlorodihydrofluorescein diacetate (DCFH-DA, Beyotime, Shanghai, China) and then measured by flow cytometry (Attune NxT, Thermo Fisher Scientific, USA).

### Mitochondrial membrane potential

The mitochondrial membrane potential was detected by using JC-1 kit (Servicebio, Wuhan, China). According to the protocols, HK-2 cells were washed twice with PBS and then stained with JC-1 at 37°C for 20 min. In the end, the cells were observed under a fluorescent microscope.

### Enzyme-linked immunosorbent assay (ELISA)

The commercial ELISA kits (Elabscience Biotechnology, Wuhan, China) were used to measure the concentrations of IL-6, TNF-α, and IL-1β from the supernatant of cultured HK-2 and TCMK-1 cells in different groups. The concentrations of SOD, MDA, and GSH-PX in HK-2 cells culture media were determined using commercial kits (Jiancheng, Nanjing, China) according to the manufacturers' protocols.

### Statistical analysis

All experiments in the study were repeated at least three times. Data were presented as mean ± SD in this study. Statistical significance between two groups was determined by the two-tailed student *t* test and comparison between multiple groups were analyzed by one-way analysis of variance (ANOVA). P value less than 0.05 was considered as significant. All statistical analyses were performed by GraphPad Prism 7.01 (GraphPad Software, San Diego, CA, USA).

## Results

### Isolation and characterization of platelet-derived EVs

**Figure 1:**
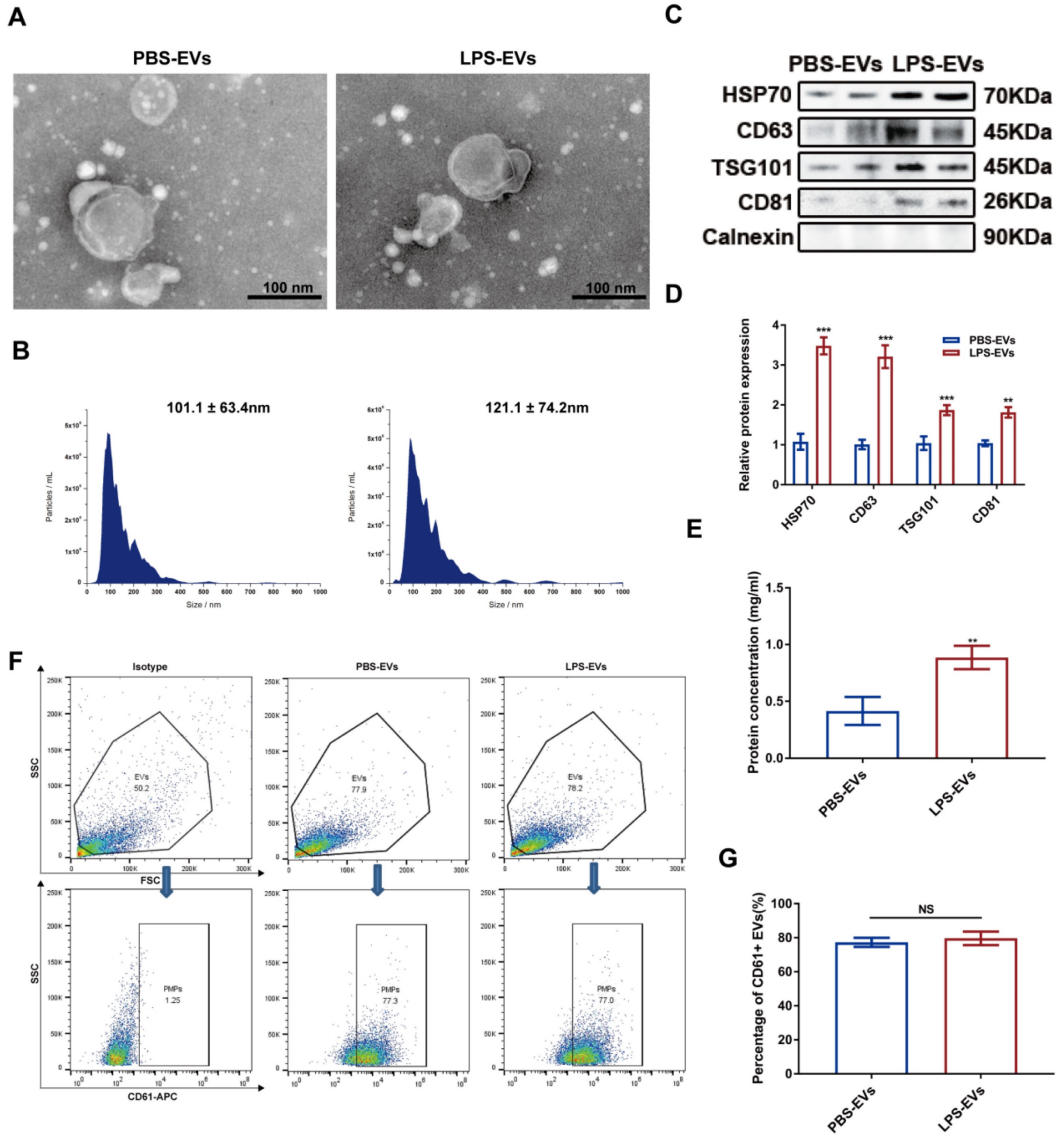
** Isolation and characterization of platelet-derived EVs.** (A) Morphology of PBS-EVs and LPS-EVs by TEM (scale bar, 100 nm). (B) The size distribution of PBS-EVs and LPS-EVs was measured by NTA. (C-D) Western blotting and densitometry analysis of HSP70, CD63, TSG101, CD81, and Calnexin in PBS-EVs and LPS-EVs (20 μg, n = 3). (E) The protein concentration of PBS-EVs and LPS-EVs derived from 1 ml of platelets supernatant was determined by BCA assay (n = 3). (F) The flow cytometry analysis of the proportion of platelets in PBS-EVs and LPS-EVs was identified by staining with CD61-APC (n = 3). (G) Quantitative analysis of percentage of CD61 positive EVs (SSC, side scatter; FSC, forward scatter). **P < 0.01, ***P < 0.001.

Platelet-derived EVs were extracted from same supernatant (1 ml) of PBS or LPS-stimulated platelets. Both PBS-EVs and LPS-EVs derived from platelets showed typical morphology of EVs under TEM (Figure 1A). The average size of the EVs purified from platelets treated with PBS or LPS were 101.1 ± 63.4 nm and 121.1 ± 74.2 nm, respectively (Figure 1B). Western blot analysis showed that these EVs were positive for HSP70, CD63, TSG101, and CD81 but were negative for Calnexin. Moreover, LPS-treated platelets secreted more EVs than PBS control (Figure 1C-D), which was further confirmed by the results from BCA protein assay (Figure 1E). To determine the proportion of PBS-EVs and LPS-EVs derived from platelets, we subjected EVs to flow cytometry analysis to detect CD61 expression, which is a platelet marker. There was no statistical significance in platelet origin (77.27 ± 1.53 vs 79.67 ± 2.78, *p* = 0.431) between two EVs groups (Figure 1F-G).

**Figure 2:**
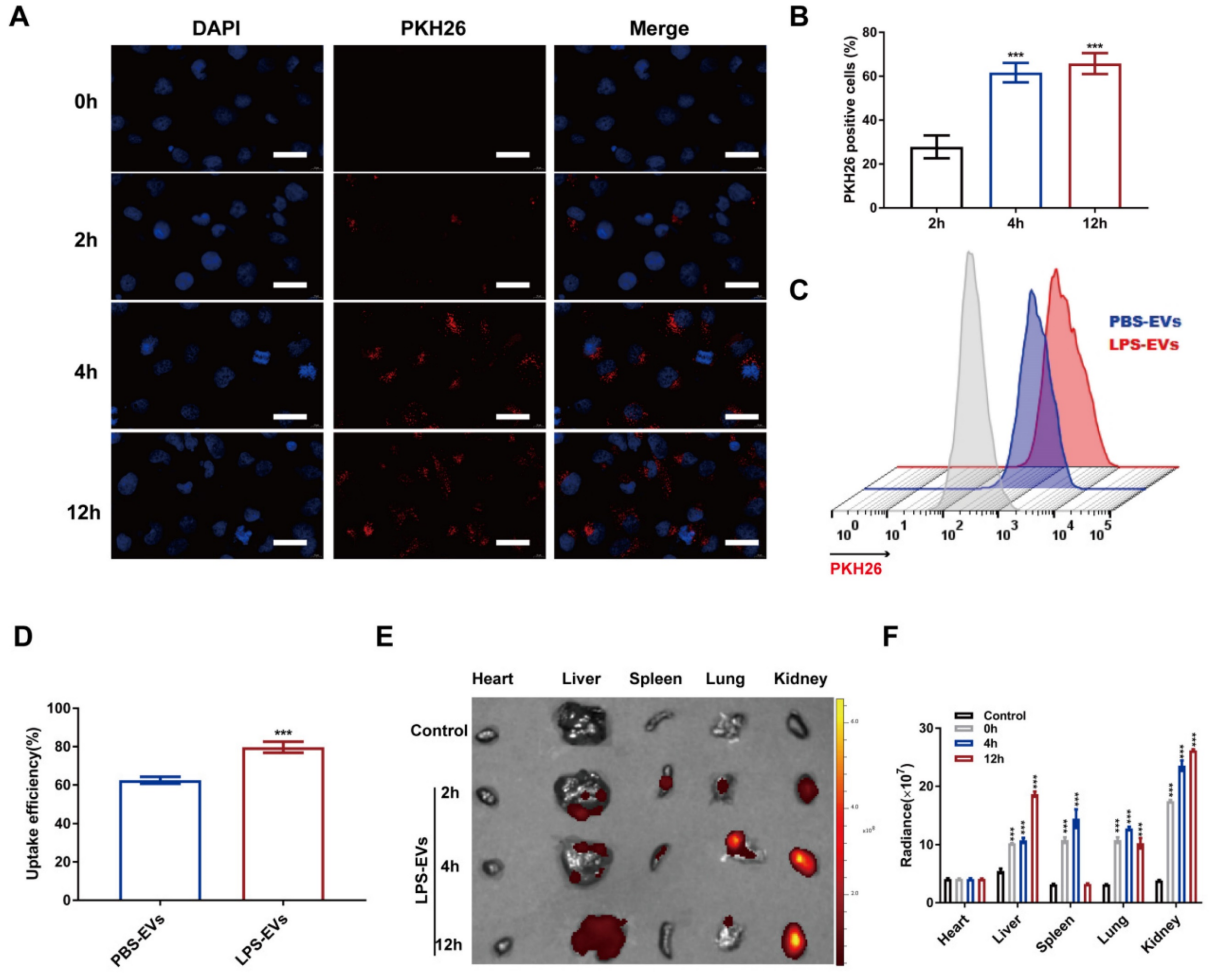
** Platelet-derived EVs homing to kidneys.** (A) Representative confocal microscope images of co-culture of LPS-EVs labeled by PKH26 with HK-2 cells (scale bar, 10 μm). (B) Quantitative analysis of PKH26 positive cells at indicated times (n = 3). (C) Flow cytometry analysis of uptake efficiency of PBS-EVs and LPS-EVs *in vitro*. (D) Quantitative analysis of cellular uptake of PBS-EVs and LPS-EVs in HK-2 cells (n = 3). (E) Representative biodistribution images of radiance in organs (heart, liver, spleen, lung, and kidney) at indicated times. (F) Quantitative analysis of *in vivo* uptake of LPS-EVs (n = 3). ***P < 0.001.

Next, to examine the uptake of platelet-derived EVs by RTECs *in vitro*, PKH26-labeled LPS-EVs (100 μg) were subjected to HK-2 cells. Confocal microscopy indicated that LPS-EVs were absorbed by HK-2 cells at 2 h. Moreover, HK-2 cells showed increased uptake efficiency with an extended time of co-incubation with LPS-EVs. However, the internalization of LPS-EVs was not significantly higher at 12 h than that at 4 h (*p* = 0.333) (Figure 2A-B). Meanwhile, flow cytometry analysis revealed an increased internalization of LPS-EVs than PBS-EVs in HK-2 cells after 4-h co-incubation (79.73 ± 2.86 vs 62.50 ± 1.75, *p* < 0.001) (Figure 2C-D). Similarly, bioluminescence imaging of the dissected organs showed that LPS-EVs accumulated in the kidney at 2 h and retained within the kidney 12 h after intravenous injection of PKH26-labeled LPS-EVs (100 μg/mouse) (Figure 2E-F). Thus, we finally selected 100 μg LPS-EVs or PBS-EVs to inject mice 4 h before CLP in the following experiments. The above results showed that LPS stimulated platelets to release EVs and these EVs could home to kidneys.

### LPS-EVs aggravated CLP-induced kidney injury

**Figure 3:**
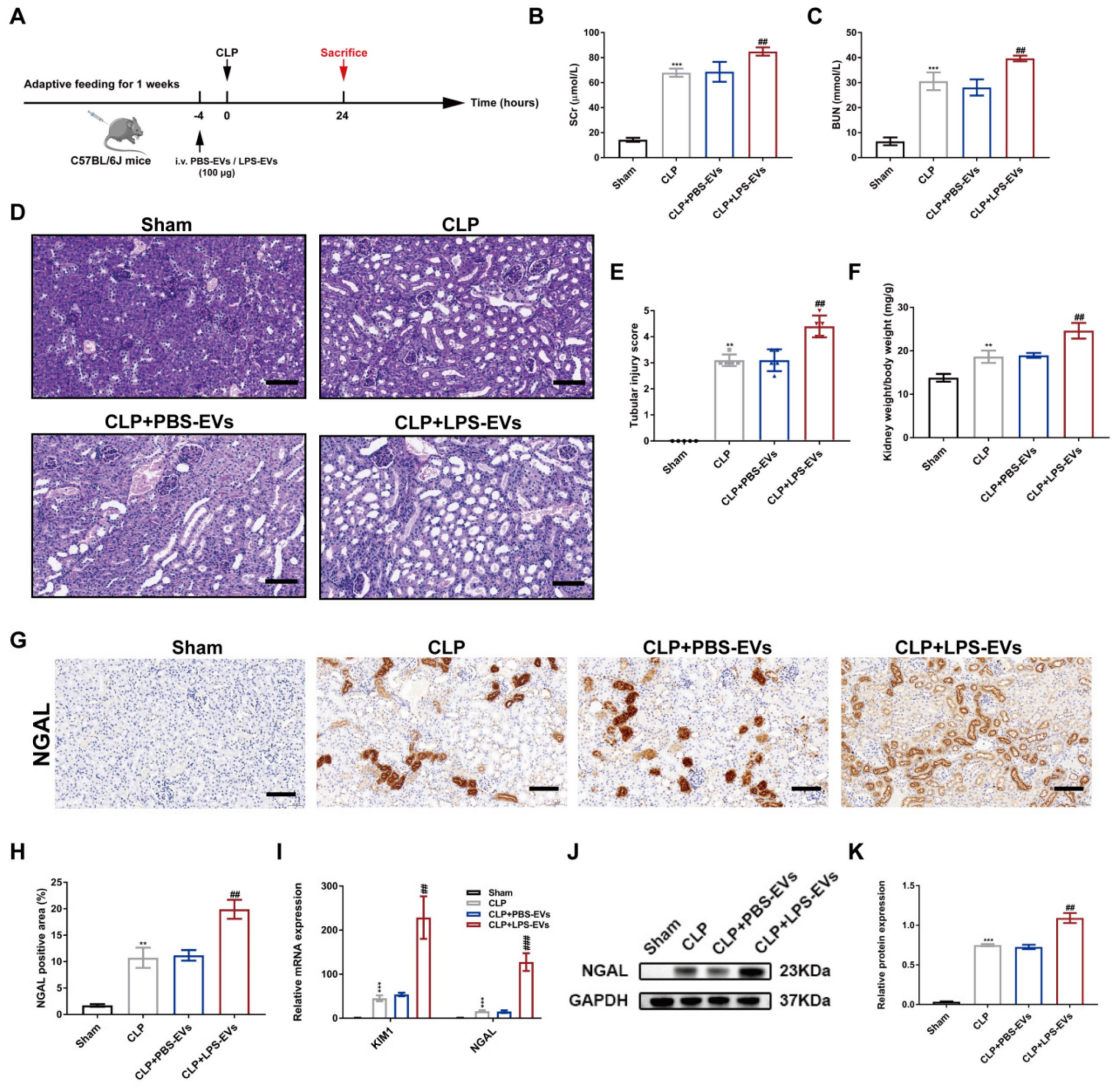
** LPS-EVs aggravated CLP-induced kidney injury.** (A) The animal protocol schematic for infusion of PBS-EVs or LPS-EVs to CLP mice. (B-C) Levels of SCr and BUN in different groups (n = 5). (D-E) Representative images of PAS staining and tubular injure score in different groups (n = 5; scale bar, 50 μm). (F) The index of kidney weight to body weight (n = 5). (G-H) Representative immunohistochemistry images and quantitative analysis of NGAL positive areas in kidney tissue sections (n = 5; scale bar, 50 μm). (I) Quantitative real-time PCR analysis of the mRNA expression of KIM1 and NGAL in kidneys (n = 3). (J-K) Western blotting and densitometry analysis of NGAL in kidneys (n = 3). **P < 0.01, ***P < 0.001; ^##^P < 0.01, ^###^P < 0.001.

To further clarify the role of platelet-derived EVs in septic AKI, we transfused PBS-EVs or LPS-EVs into the CLP mice. The animal protocol schematic was shown in Figure 3A. CLP induced significant renal dysfunction as revealed by increased levels of SCr and BUN (Figure 3B-C). In addition, LPS-EVs pretreatment aggravated kidney injury compared to PBS-EVs, which is characterized by dilation, swelling, tubular formation and absence of brush border of the proximal tubules associated with increased of levels of SCr, BUN and the ration of kidney weight to body weight (Figure 3D-F). As indicated in Figure 3G-H, neutrophil gelatinaseassociated lipocalin (NGAL) was mainly upregulated in renal tubules following CLP and LPS-EVs administration further increased NGAL expression. Meanwhile, in comparison to PBS-EVs, LPS-EVs transfusion increased mRNA expression of kidney injury molecule 1 (KIM1) and NGAL (Figure 3I), as well as protein level of NGAL in CLP injured kidneys (Figure 3J-K). Taken together, these data indicated that LPS-EVs exacerbated septic AKI.

### LPS-EVs promoted inflammation and apoptosis during septic AKI

**Figure 4:**
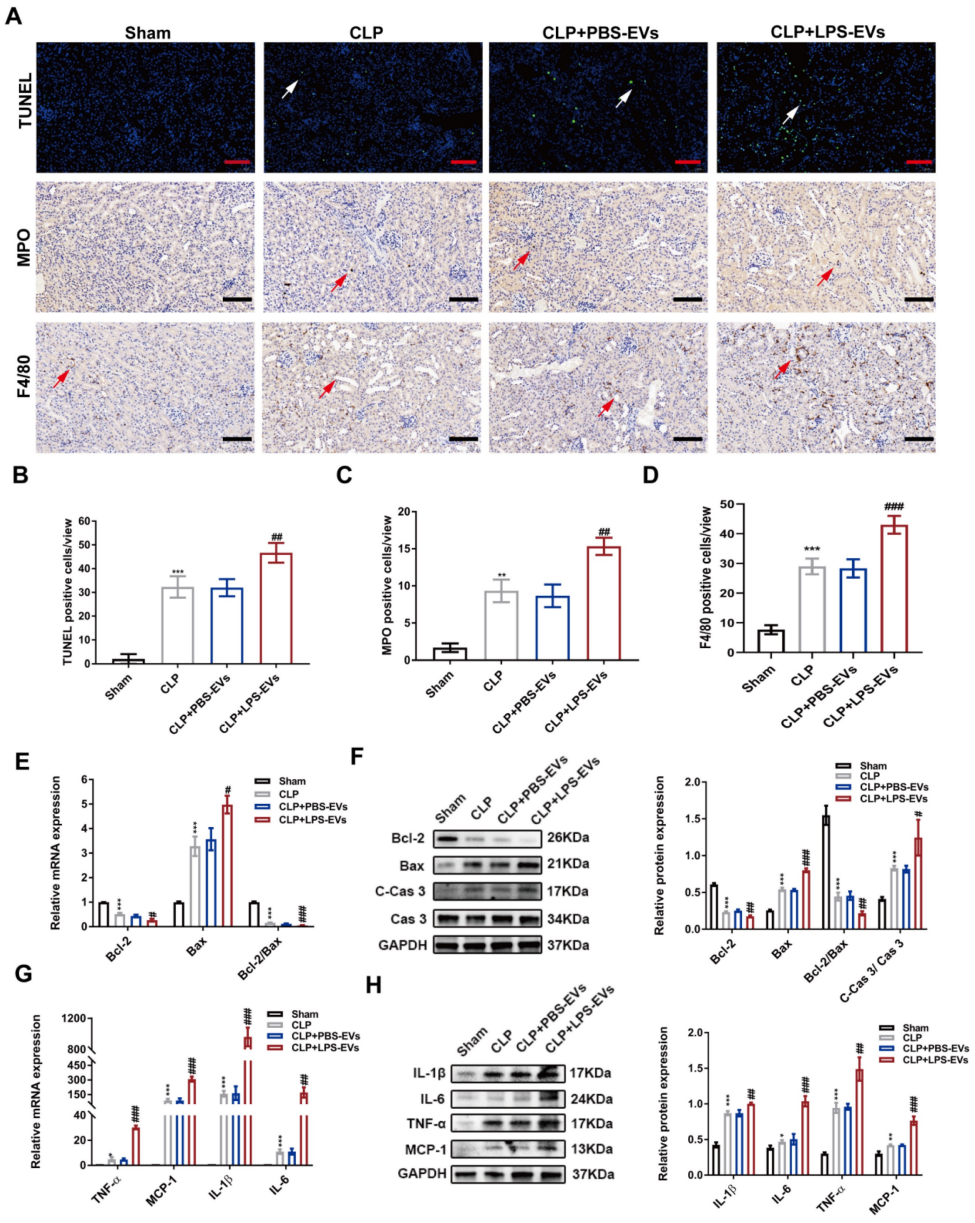
** LPS-EVs promoted kidney inflammation and apoptosis in CLP-injured kidneys.** (A) Representative TUNEL, MPO and F4/80 staining images in kidneys (scale bar, 50 μm). (B) Quantitative analysis of TUNEL positive cells in kidneys (n = 5). (C) Quantitative analysis of MPO positive cells in kidneys (n = 5). (D) Quantitative analysis of F4/80 positive cells in kidneys (n = 5). (E) The mRNA expression of Bcl-2, Bax and Bcl-2/Bax in kidneys analyzed by quantitative real-time PCR (n = 3). (F) Western blotting and quantification by densitometry of Bcl-2, Bax, Cas 3, and C-Cas 3 in kidneys (n = 3). (G) The mRNA expression of IL-6, MCP-1, TNF-α and IL-1β in kidneys analyzed by quantitative real-time PCR (n = 3). (H) Western blotting and densitometry analysis of the protein levels of IL-6, MCP-1, TNF-α and IL-1β in kidneys (n = 3).

Inflammation and apoptosis in kidneys are considered as hallmarks of septic AKI 7,8. Thus, we concentrated on the effect of platelet-derived EVs on inflammation and apoptosis during septic AKI *in vivo* and *in vitro*. Compared with PBS-EVs, LPS-EVs significantly increased the number of TUNEL positive cells (Figure 4A-B) in CLP injured kidneys. Immunohistochemistry staining of kidney tissues revealed that LPS-EVs transfusion promoted neutrophils infiltration indicated by MPO staining (Figure 4A, C). In addition to neutrophils, we also observed increased recruitment of macrophages (F4/80 positive cells) in CLP mice treated with LPS-EVs compared to PBS-EVs (Figure 4D). Moreover, LPS-EVs injection upregulated pro-apoptotic markers of Cleaved-Caspase 3 (C-Cas 3) and Bax along with downregulated anti-apoptotic markers of Bcl-2 (Figure 4E-F). Also, LPS-EVs administration significantly increased pro-inflammatory markers of MCP-1, TNF-α, IL-1β, and IL-6 in CLP injured kidneys (Figure 4G-H).

**Figure 5:**
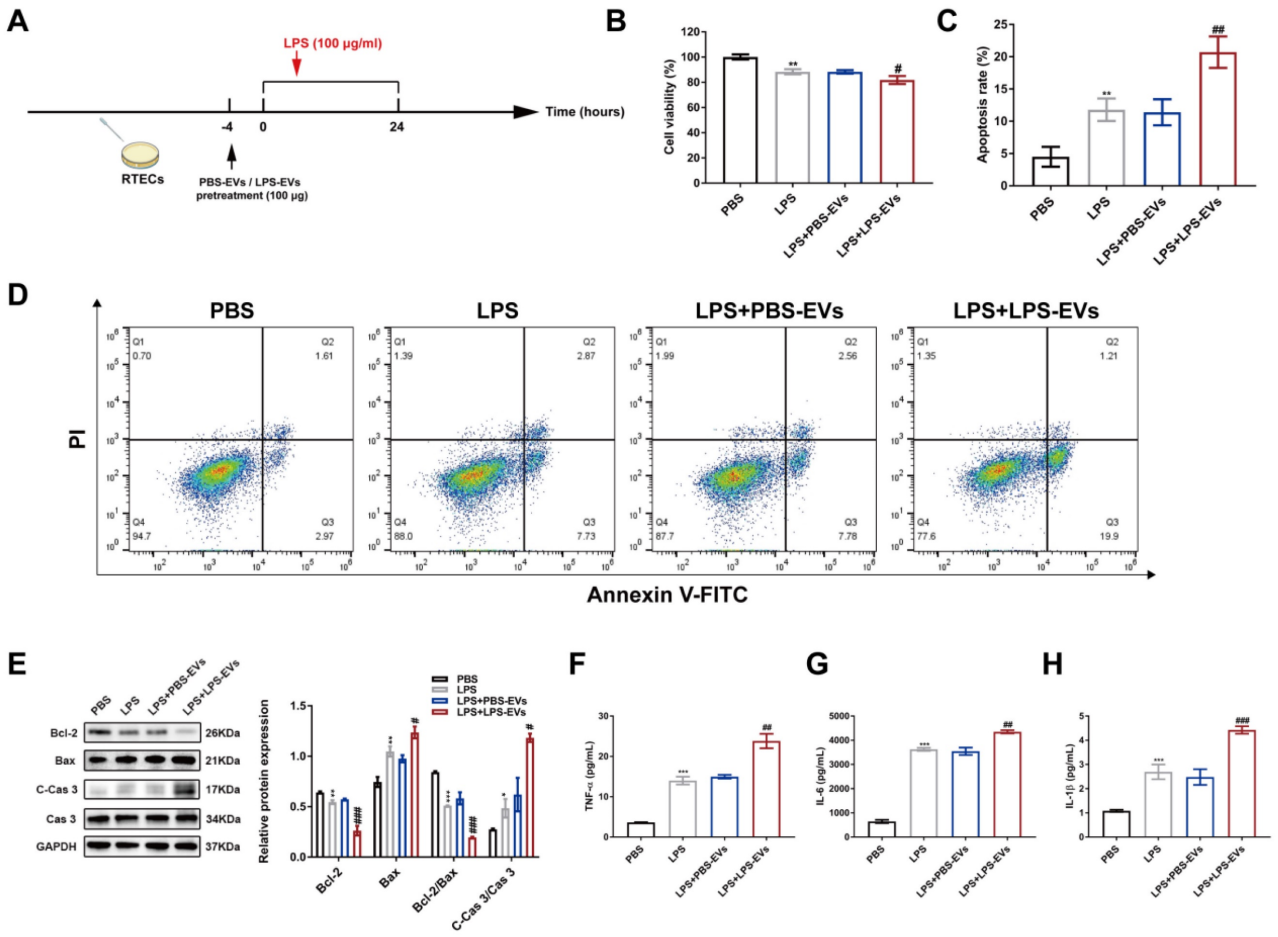
** LPS-EVs promoted inflammation and apoptosis in LPS-stmulated HK-2 cells.** (A) The cellular protocol schematic for administration of PBS-EVs or LPS-EVs to LPS-stimulated HK-2 cells. (B) The cytotoxic effect of PBS-EVs or LPS-EVs on HK-2 cells was determined by CCK8 assay (n = 6). (C-D) Representative flow cytometric plots and quantitative analysis of apoptosis rate of HK-2 cells and in different groups (n = 3). (E) Western blotting and densitometry analysis of protein levels of Bcl-2, Bax, Cas 3, and C-Cas 3 in HK-2 cells (n = 3). (F-H) The concentrations of TNF-α, IL-6, and IL-1β in the supernatant of HK-2 cells (n = 6). *P < 0.05, **P < 0.01, ***P < 0.001; ^#^P < 0.05, ^##^P < 0.01, ^###^P < 0.001.

To mimic the sepsis *in vitro*, we used LPS with different concentrations (0-100 μg/ml) to stimulate HK-2 cells. As delineated in Figure S1, the cell viability was inhibited and apoptosis rate was increased in HK-2 cells in a dose-dependent manner. Thus, we decided to treat HK-2 cells with 100 μg/ml LPS for 24 h *in vitro.* The cellular protocol schematic was shown in Figure 5A. HK-2 cells were pretreated with 100 μg PBS-EVs or LPS-EVs 4 h before LPS stimulation. Compared with PBS-EVs, LPS-EVs promoted cellular injury as indicated by an inhibition of cell vaibility (Figure 5B) associated with an increased apoptosis rate in LPS-stimulated HK-2 cells (Figure 5C-D). Besides, LPS-EVs administration increased protein levels of C-Cas 3 and Bax and decreased Bcl-2 protein compared to PBS-EVs in HK-2 cells (Figure 5E). The levels of TNF-α, IL-6 together with IL-1β in the supernatant were considerably increased after LPS-EVs treatment in contrast to PBS-EVs (Figure 5F-H). However, supernatant depleted of LPS-EVs showed no impact on cell apoptosis (Figure S2).

To better demonstrate the harmful effect of platelet-derived EVs, the impact of each EVs was further evaluated *in vivo* and *in vitro*. We injected 100 μg PBS-EVs or LPS-EVs to WT mice. Similarly, compared to PBS-EVs group, mice received LPS-EVs injection displayed marked kidney damage with tubular dilation, swelling and lost of brush border, as indicated by PAS staining (Figure S3A-B). Meanwhile, LPS-EVs treatment significantly exacerbated renal function as increased the levels of SCr and BUN (Figure S3C-D). Moreover, LPS-EVs upregulated the mRNA expression of KIM1 and NGAL in the kidneys of WT mice (Figure S3E). Also, in comparison with PBS-EVs, LPS-EVs increased the protein level of NGAL (Figure S3F). In addition, *in vitro* experiments showed that LPS-EVs caused cellular injury by inhibiting the cell viability and increasing the cell apoptosis (Figure S3G-I). Consistently, LPS-EVs upregulated the protein levels of C-Cas 3 and Bax while downregulated Bcl-2 (Figure S3J). Furthermore, we found the levels of TNF-α, IL-6, and IL-1β in the supernatant of HK-2 cells were dramatically induced by LPS-EVs (Figure S3K-M). Collectively, these results suggested that LPS-EVs exacerbated septic AKI via promoting apoptosis and inflammation.

### LPS-EVs promoted oxidative stress and mitochondrial dysfunction in septic AKI

**Figure 6:**
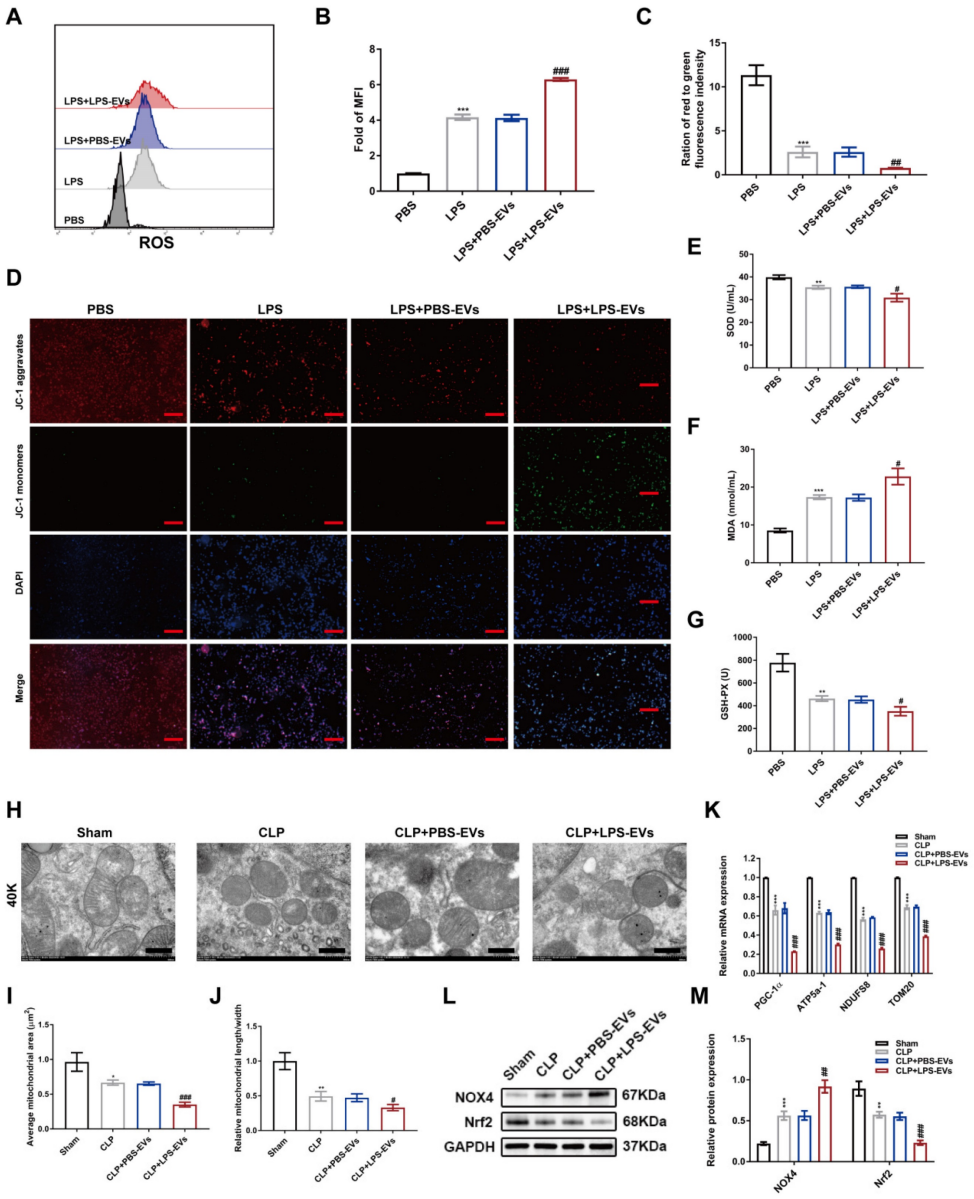
** LPS-EVs promoted oxidative stress and mitochondrial dysfunction in septic AKI.** (A) ROS production in HK-2 cells was detected by flow cytometry. (B) Quantitative analysis of mean fluorescence intensity (MFI) of ROS in HK-2 cells (n = 3). (C-D) Representative images of mitochondrial membrane potential by JC-1 staining and quantification analysis of the ratio of red to green immunofluorescence density (n = 3; scale bar, 50 μm). (E-G) The levels of SOD, MDA, and GSH-PX in the supernatant of HK-2 cells (n = 6). (H) Representative images of mitochondrial structures by TEM (scale bar, 500 nm). (I) Quantification of average mitochondrial area in different groups (n = 3). (J) Quantification of the ratio of length to width of mitochondria in different groups (n = 3). (K) The mRNA expression of PGC-1α, ATP5a-1, NDUFS8, and TOM20 in kidneys was analyzed by quantitative real-time PCR (n = 3). (L-M) Western blotting and densitometry analysis of the protein levels of NOX4 and Nrf2 in kidneys (n = 3). *P < 0.05, **P < 0.01, ***P < 0.001; ^#^P < 0.05, ^##^P < 0.01, ^###^P < 0.001.

Recent studies have identified that mitochondrial damage and oxidative stress are critical drivers of AKI 34,35. First, to examine the level of ROS, HK-2 cells were incubated with DCFH-DA. As displayed in Figure 6A-B, flow cytometry analysis showed that LPS stimulation increased ROS production in HK-2 cells, and LPS-EVs further enhanced ROS accumulation compared to PBS-EVs. Loss of mitochondrial membrane potential is reported to be associated with accumulation of ROS 36. Then, we decided to detect the mitochondrial membrane potential by using JC-1. LPS stimulation damaged the membrane potential of mitochondria, and LPS-EVs aggravated collapse of membrane potential in LPS-stimulated HK-2 cells (Figure 6C-D). Meanwhile, we observed a significant reduction in SOD and GSH-PX levels, as well as an increased production of MDA in HK-2 cells stimulated with LPS and LPS-EVs (Figure 6E-G).

To further illustrate the effect of LPS-EVs *in vivo*, we observed the renal mitochondrial morphology by TEM. Mitochondrial damages including mitochondrial swelling, mitochondrial membrane rupture and absence of cristae were evident in CLP mice. Compared to PBS-EVs, LPS-EVs significantly impaired mitochondrial function as revealed by decreased mitochondrial area, mitochondrial length to width ratio and mRNA expressions of PGC-1α, ATP5a-1, NDUFS8, and TOM20 (Figure 6H-K). Nicotinamide adenine dinucleotide phosphate (NADPH) oxidase 4 (NOX4) is expressed in the kidney and contributes to the production of ROS 37. While the transcription factor nuclear factor erythroid 2-related factor 2 (Nrf2) mediates downstream antioxidants in response to oxidative stress 38. Then, the levels of NOX4 and Nrf2 were examined via exploiting western blotting. The results showed that the level of NOX4 was raised, while Nrf2 was downregulated in CLP injured kidneys. More importantly, CLP mice treated with LPS-EVs further increased the protein level of NOX4 and decreased Nrf2, compared to those receiving PBS-EVs (Figure 6L-M). Overall, these data indicated that LPS-EVs promoted oxidative stress and mitochondrial dysfunction in septic AKI.

### Infusion with plasma EVs from CLP mice exacerbated renal function

To better reflect the diseased conditions *in vivo*, plasma EVs from CLP mice were used to validate the findings from LPS-EVs. The purified Sham-EVs and CLP-EVs were characterized with typical bilayer membrane under TEM (Figure S4A). NTA analysis showed that both Sham-EVs and CLP-EVs were around 100 nm in diameter (Figure S4B). Notably, CLP-induced mice secreted more EVs compared to sham mice, as indicated by increased particle concentration and protein concentration from equal plasma volumes (1 ml) (Figure S4C-D). Moreover, western blot analysis showed higher expressions of HSP70, CD63, TSG101, and CD81 in CLP-EVs than Sham-EVs. However, no significant difference was found in platelet origin between Sham-EVs and CLP-EVs by staining with CD61 and then detected by flow cytometry (81.47 ± 1.66 vs 81.17 ± 2.79, *p* = 0.881) (Figure S4G-H).

Then, *in vivo* experiments, WT mice were injected with Sham-EVs and CLP-EVs (100 μg/mouse i.v) for 4 h (Figure S5A). As illustrated in Figure S5B-E, CLP-EVs caused severe renal dysfunction and tubular damage as indicated by increased levels of SCr, BUN and tubular injury scores. In addition, the mRNA expression of kidney injury markers of KIM1 and NGAL was significantly upregulated after CLP-EVs administration (Figure S5F) associated with increased protein level of NGAL (Figure S5G). Likewise, further results indicated that CLP-EVs exacerbated renal function via promoting apoptosis (Figure S5H-I, K-L) and enhancing inflammation in kidneys (Figure S5H, J, M-N). Also, *in vitro* experiments revealed that transfusion of CLP-EVs to TCMK-1 cells (Figure S6A) caused cellular injury (Figure S6B) via promoting apoptosis (Figure S6C-E) and inflammation (Figure S6F-G). In general, all these findings revealed that plasma EVs from CLP mice impaired kidney function via promoting cell apoptosis and inflammation.

### ARF6 derived from LPS-EVs exacerbated septic AKI

**Figure 7:**
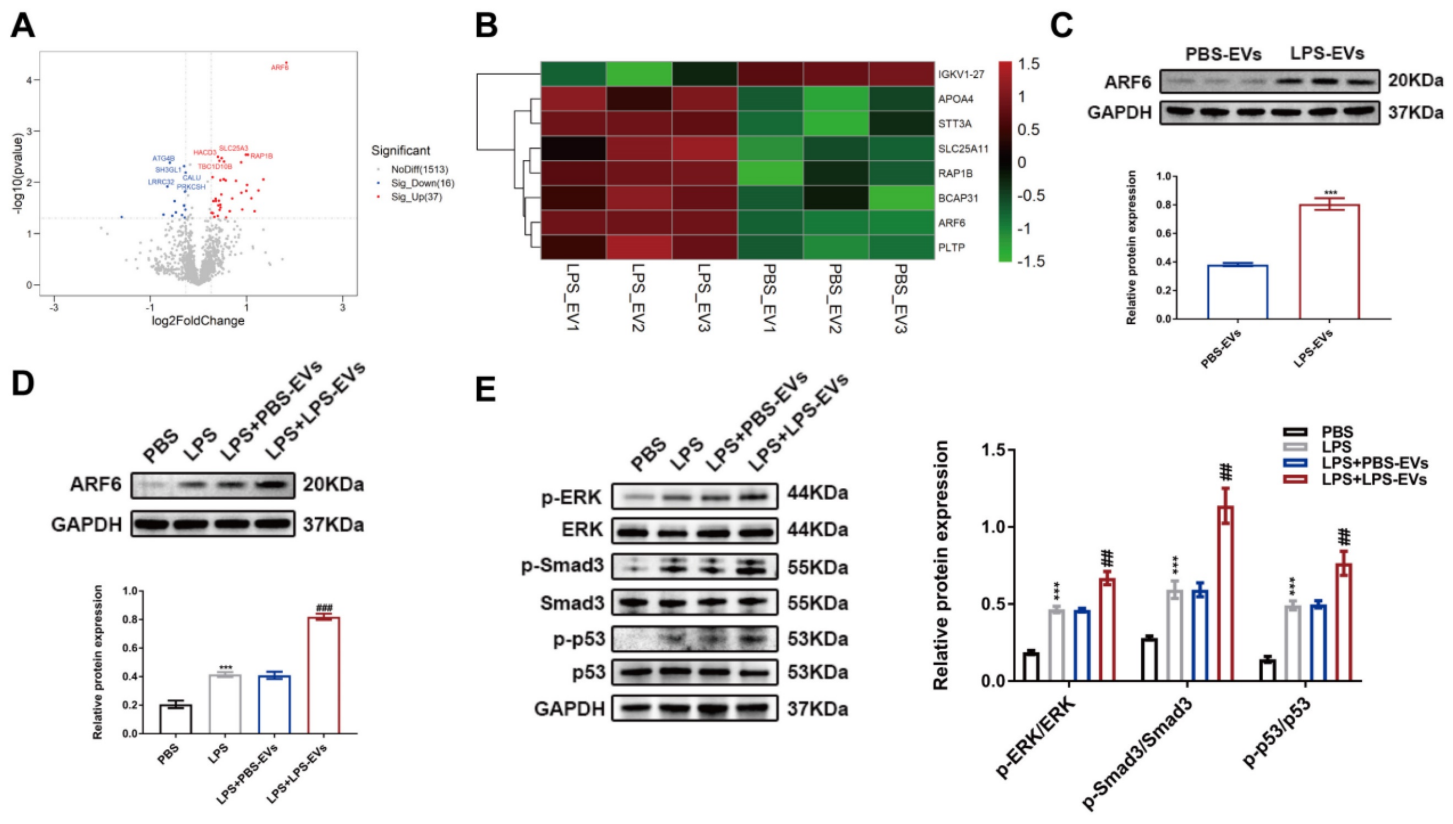
** ARF6 enriched in LPS-EVs activated ERK, Smad3 and p53.** (A) The volcano map of differentially expressed proteins (DEPs) between LPS-EVs and PBS-EVs. (B) The heat map of DEPs between LPS-EVs and PBS-EVs. (C) Western blotting and densitometry analysis of ARF6 between PBS-EVs and LPS-EVs (n = 3). (D) Western blotting and densitometry analysis of ARF6 level in HK-2 cells (n = 3). (E) Western blotting and densitometry analysis of the protein levels of p-ERK, ERK, p-Smad3, Smad3, p-p53, and p53 in HK-2 cells (n = 3). ***P < 0.001; ^##^P < 0.01, ^###^P < 0.001.

In order to better elucidate the role of platelet-derived EVs in septic AKI, we performed quantitative proteomics analysis to screen different expressed proteins between PBS-EVs and LPS-EVs. As shown in Figure 7A-B, ARF6 was the most abundant protein in LPS-EVs compared to PBS-EVs (fold change = 3.55). As ARF6 was the most abundant protein in LPS-EVs, and previous studies have reported that ARF6 regulates inflammation, oxidative stress and apoptosis in various disease 39-42. Thus, we focused on ARF6 in the following experiments and explored the role of ARF6 in septic AKI. Western blotting confirmed that LPS-EVs contained high protein level of ARF6 than PBS-EVs (Figure 7C), and that endogenous ARF6 protein was significantly upregulated in HK-2 cells after LPS treatment. Moreover, LPS-stimulated HK-2 cells showed upregulated protein level of ARF6 after co-incubation with LPS-EVs when compared to PBS-EVs (Figure 7D). Previously, it has been found that MAPKs play vital roles in regulating septic AKI via activating p53 and NOX4 16. Meanwhile, ARF6 could activate ERK signal pathway that is responsible for induction of NOX4 expression 43,44. Interestingly, we detected upregulated levels of phosphorylated ERK, Smad3, and p53 in HK-2 cells with LPS treatment, and LPS-EVs further enhanced these protein levels (Figure 7E).

**Figure 8:**
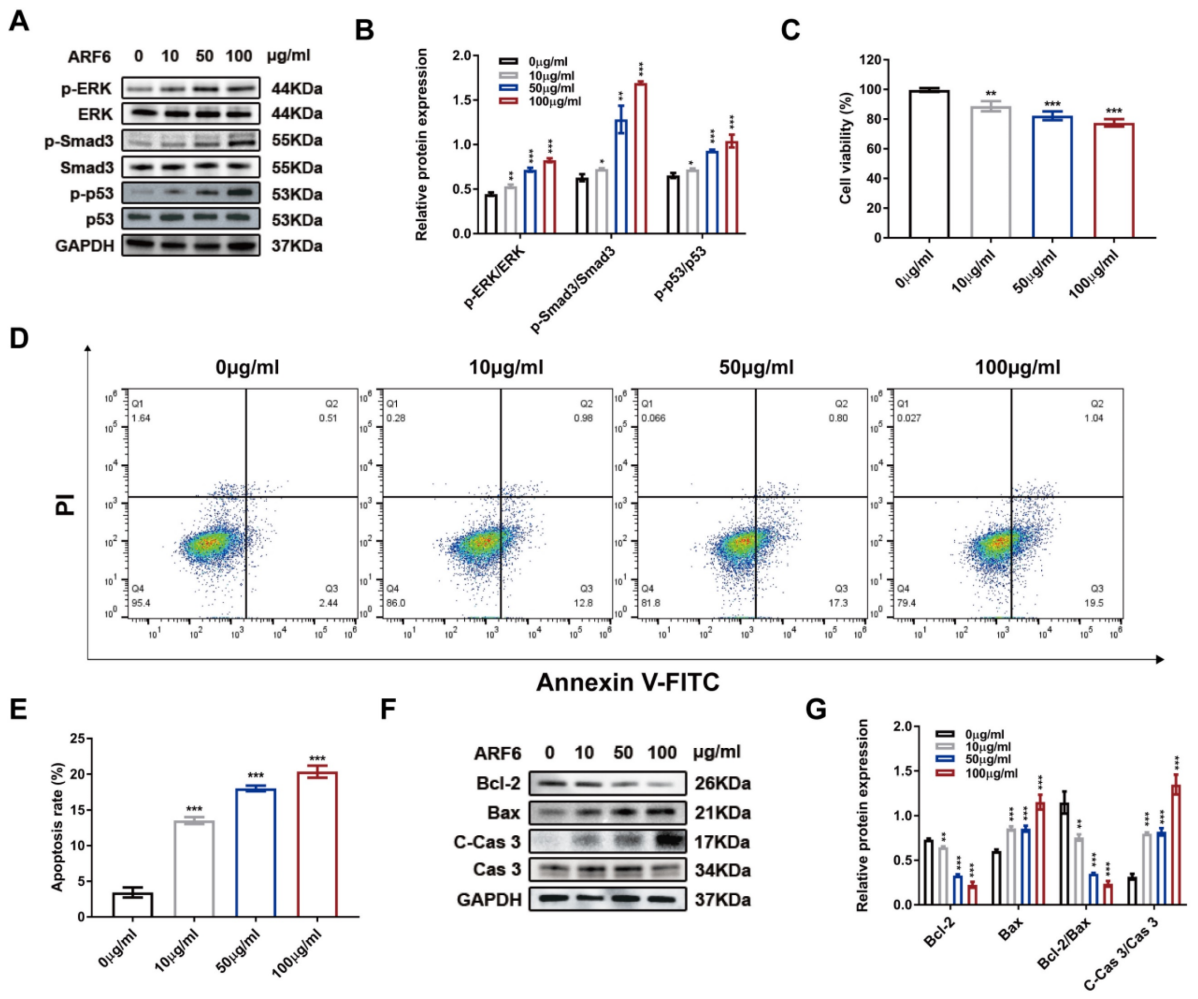
** LPS-EVs derived ARF6 contributed to cellular injury.** HK-2 cells were treated with recombinant ARF6 (0, 10, 50, or 100 μg/ml) for 24 hours. (A) Western blotting and (B) densitometry analysis of protein levels of p-ERK, ERK, p-Smad3, Smad3, p-p53, and p53 in HK-2 cells (n = 3). (C) The cytotoxic effect of recombinant ARF6 (0, 10, 50, and 100 μg/ml) on HK-2 cells determined by CCK8 assay (n = 6). (D) Representative flow cytometric plots of apoptosis of HK-2 cells stimulated by recombinant ARF6 (0, 10, 50, and 100 μg/ml). (E) Quantitative analysis of apoptosis rate of HK-2 cells (n = 3). (F-G) Western blotting and densitometry analysis of the protein levels of Bcl-2, Bax, Cas 3, and C-Cas 3 in HK-2 cells (n = 3). **P < 0.01, ***P < 0.001.

Subsequently, to determine whether the abundant ARF6 could recapitulate the effect of LPS-EVs during septic AKI, we used recombinant ARF6 protein to stimulate HK-2 cells. As presented in Figure 8A-B, ARF6 increased the phosphorylation of ERK, Smad3 and p53 in HK-2 cells in a dose-dependent manner. Moreover, recombinant ARF6 protein also caused cellular injury and increased cell apoptosis (Figure 8C-E). As revealed by western blotting, recombinant ARF6 was able to upregulate protein levels of C-Cas 3 and Bax and downregulate Bcl-2 in HK-2 cells, indicating that ARF6 was responsible for promoting septic AKI in LPS-EVs (Figure 8F-G).

### LPS-upregulated ARF6 via TLR4 activation in HK-2 cells

**Figure 9:**
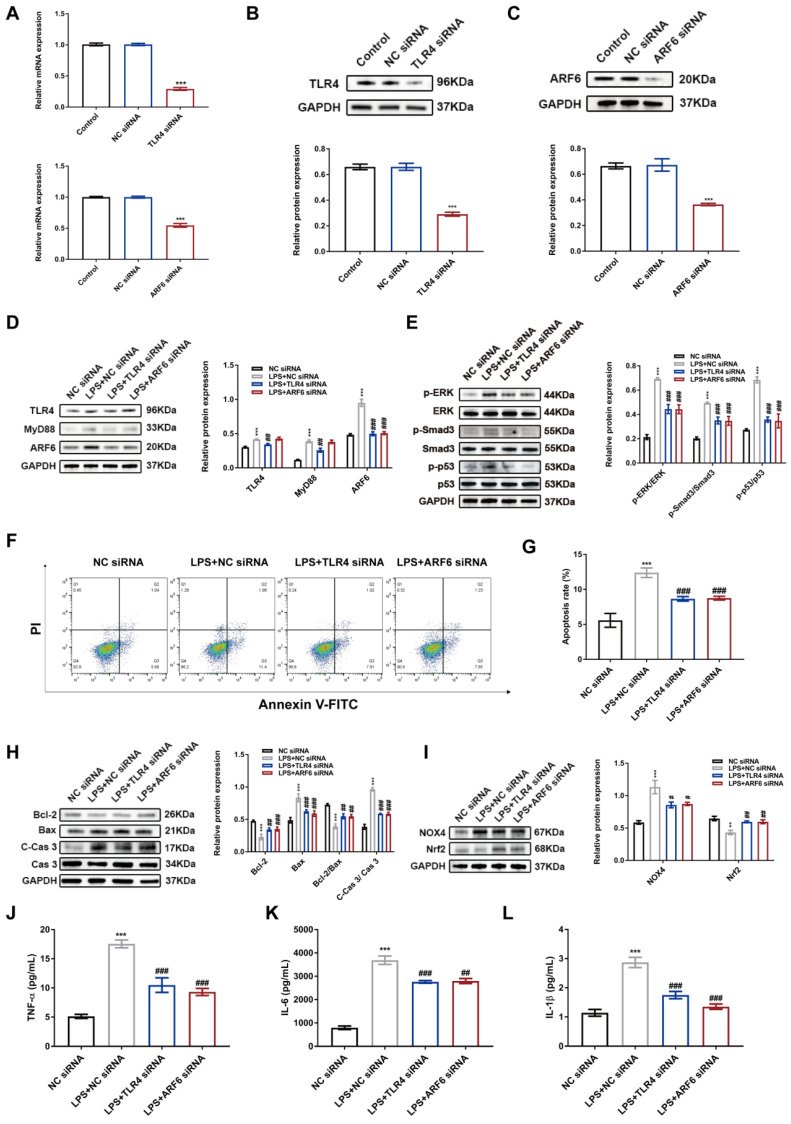
** LPS-stimulated upregulation of ARF6 was mediated by TLR4 signaling in HK-2 cells.** HK-2 cells were transfected with siRNAs and then stimulated with LPS (100 μg/ml) for 24 h. (A) The mRNA expression of TLR4 and ARF6 in HK-2 cells was analyzed by quantitative real-time PCR (n = 3). (B-C) Western blotting and densitometry analysis of TLR4 and ARF6 in HK-2 cells (n = 3). (D) Western blotting and quantification by densitometry of the protein levels of TLR4, MyD88, and ARF6 in HK-2 cells with TLR4 or ARF6 knockdown after LPS stimulation (n = 3). (E) Western blotting and densitometry analysis of p-ERK, ERK, p-Smad3, Smad3, p-p53, and p53 with TLR4 or ARF6 knockdown after LPS stimulation (n = 3). (F) Representative flow cytometric plots of apoptosis of HK-2 cells. (G) Quantitative analysis of apoptosis rate in HK-2 cells with TLR4 or ARF6 knockdown after LPS stimulation (n = 3). (H) Western blotting and densitometry analysis of Bcl-2, Bax, Cas 3, and C-Cas 3 in HK-2 cells (n = 3). (I) Western blotting and densitometry analysis of NOX4 and Nrf2 in HK-2 cells (n = 3). (J-K) The concentrations of TNF-α, IL-6, and IL-1β in the supernatant of HK-2 cells (n = 6). **P < 0.01, ***P < 0.001; ^#^P < 0.05, ^##^P < 0.01, ^###^P < 0.001.

TLR4/MyD88 axis is an important intracellular mediator to regulate inflammation and cell death in septic AKI 14,15,45. Meanwhile, we found an upregulated protein level of ARF6 after LPS stimulation in HK-2 cells. Therefore, we determined whether TLR4/MyD88/ARF6 signaling promoted septic AKI. We transfected HK-2 cells with TLR4 and ARF6 siRNAs and subjected transfected cells to LPS stimulation, and suppressed TLR4 and ARF6 expression was observed (Figure 9A-C). LPS-induced expression of TLR4, MyD88 and ARF6 in HK-2 cells was reversed by TLR4 siRNA, while ARF6 knockdown only affected ARF6 expression without affecting TLR4 and MyD88 expression (Figure 9D), indicating that ARF6 was regulated by TLR4 and MyD88. Moreover, both TLR4 and ARF6 knockdown reversed the upregulated expression of phosphorylation of ERK, Smad3 and p53 in HK-2 cells after LPS stimulation (Figure 9E). Flow cytometr**y** results demonstrated decreased apoptosis rate by TLR4 and ARF6 knockdown in HK-2 cells against LPS stimulation (Figure 9F-G). Meanwhile, western blotting showed that LPS upregulated proapoptotic and oxidative stress markers were reversed by TLR4 and ARF6 knockdown in HK-2 cells (Figure 9H-I). In the supernatant of HK-2 cells, the expression of pro-inflammatory cytokines was also reversed by TLR4 and ARF6 inhibition after LPS stimulation (Figure 9J-K). In general, ARF6 was upregulated by LPS via TLR4, and downregulation of ARF6 protected HK-2 cells against LPS by inhibiting apoptosis, inflammation, and oxidative stress.

### Pharmacologic inhibition of ARF6 alleviated CLP-induced septic AKI

**Figure 10:**
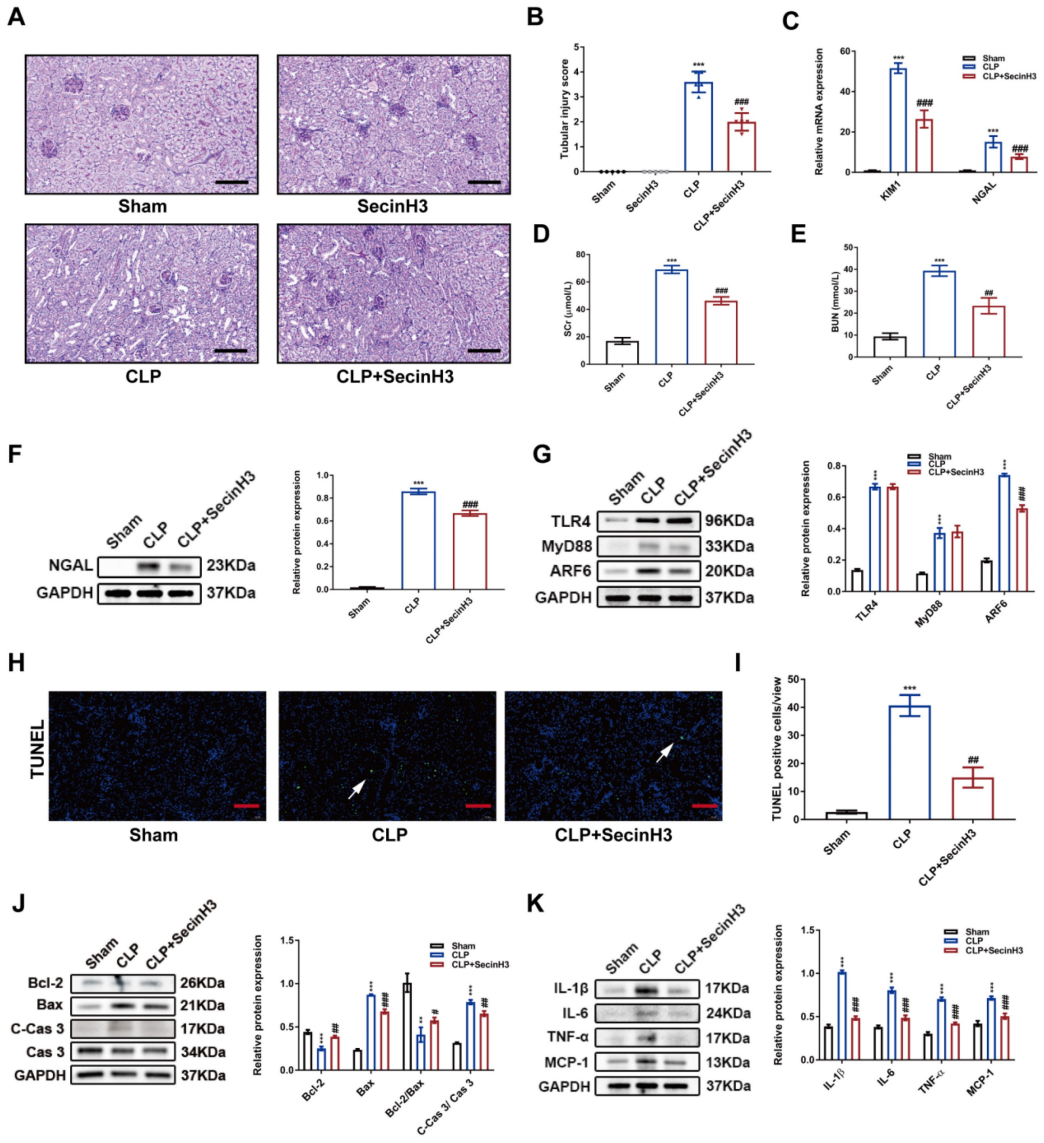
** Pharmacologic inhibition of ARF6 alleviated CLP-induced septic AKI.** SecinH3 (20 mg/kg) was intraperitoneally injected into mice 2 h before CLP to inhibit ARF6 *in vivo*. (A) Representative images of PAS staining (scale bar, 50 μm). (B) Tubular injury scores of Sham, SecinH3, CLP and CLP+SecinH3 groups (n = 5). (C) The mRNA expression of KIM1 and NGAL in kidneys was analyzed by quantitative real-time PCR (n = 3). (D) SCr and (E) BUN levels in different groups (n = 5). (F) Western blotting and densitometry analysis of NGAL in kidneys (n = 3). (G) Western blotting and densitometry analysis of TLR4, MyD88, and ARF6 in kidneys (n = 3). (H) Representative TUNEL staining images in kidneys (scale bar, 50 μm). (I) Quantitative analysis of TUNEL positive cells in kidneys (n = 5). (J) Western blotting and densitometry analysis of Bcl-2, Bax, Cas 3, and C-Cas 3 in kidneys (n = 3). (K) Western blotting and densitometry analysis of IL-6, IL-1β, TNF-α, and MCP-1 in kidneys (n = 3). **P < 0.01, ***P < 0.001; #P < 0.05, ##P < 0.01, ###P < 0.001.

To further evaluate whether ARF6 inhibitor could provide a renoprotective effect *in vivo*, we used SecinH3 (20 mg/kg) to injected mice 2 h before CLP. Pharmacologic inhibition of ARF6 remarkably alleviated septic AKI as evidenced by markedly mitigated renal morphologic damage, decreased CLP upregulated levels of SCr and BUN, and lowered mRNA expression of KIM1 and NGAL in injured kidneys (Figure 10A-E). We also observed downregulated protein level of NGAL in CLP-injured kidneys (Figure 10F). Similarly, TLR4/MyD88/ARF6 expression was also induced by CLP, and ARF6 expression was markedly inhibited by SecinH3 treatment, while no significant changes were found in TLR4 and MyD88 in CLP-injured kidneys (Figure 10G). Furthermore, SecinH3 treatment obviously inhibited the apoptosis and inflammation in CLP-injured kidneys, as revealed by the decreased apoptosis cells (Figure 10H-I) and pro-apoptotic markers (Figure 10J). We also discovered downregulated protein levels of inflammatory cytokines such as IL-1β, IL-6, TNF-α and MCP-1 in CLP-injured kidneys after SecinH3 treatment (Figure 10K). Altogether, pharmacologic inhibition of ARF6 alleviated CLP-induced septic AKI.

### Inhibition of platelet secretion alleviated septic AKI

**Figure 11:**
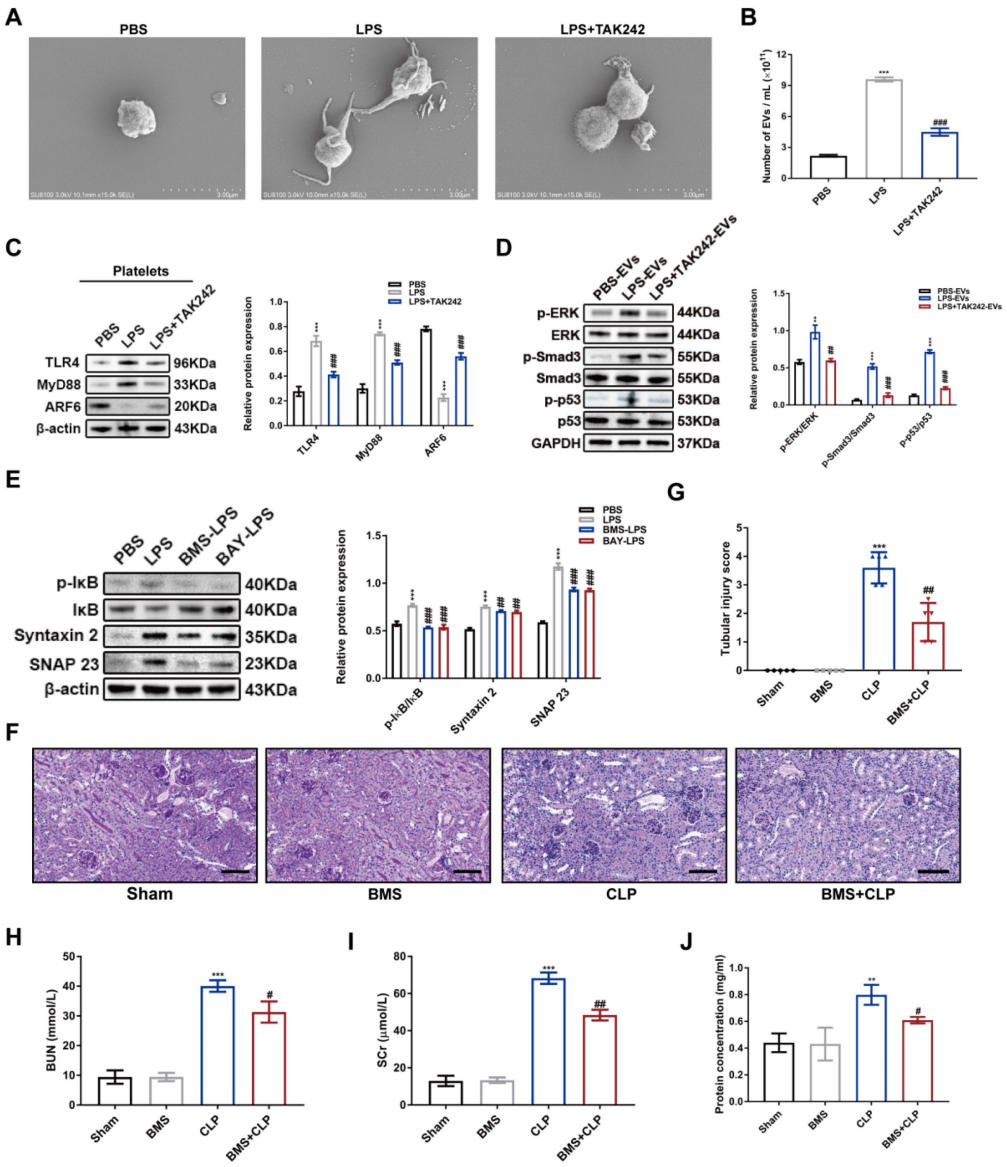
** Inhibition of platelet secretion alleviated septic AKI.** (A) The morphology of platelets treated with PBS, LPS (1 μg/ml) and LPS+TAK242 (50 mg/ml) was visualized by SEM (scale bar, 3 μm). (B) The concentration of EVs in the supernatant of platelets in PBS, LPS, and LPS+TAK242 groups was measured by NTA (n = 3). (C) Western blotting and densitometry analysis of protein levels of TLR4, MyD88, and ARF6 in platelets (n = 3). (D) Western blotting and densitometry analysis of p-ERK, ERK, p-Smad3, Smad3, p-p53, and p53 in HK-2 cells (n = 3). (E) Western blotting and densitometry analysis of p-IκB, IκB, Syntaxin 2, and SNAP 23 in HK-2 cells (n = 3). (F-G) Representative PAS staining images (scale bar, 50 μm) and tubular injury scores of Sham, BMS, CLP, and BMS+CLP groups (n = 5). (H) SCr and (I) BUN levels in Sham, BMS, CLP, and BMS+CLP groups (n = 5). (J) The protein concentration of EVs from equal plasma volumes (100 μl) in different groups was determined by BCA assay (n = 3). **P < 0.01, ***P < 0.001; ^#^P < 0.05, ^##^P < 0.01, ^###^P < 0.001.

Finally, we investigated the mechanism of platelets secretion during sepsis. Notably, scanning electron microscopy (SEM) showed that LPS-stimulated platelets were covered with membrane protrusions, which are the marks of platelet activation, degranulation, and EVs secretion. However, treatment of TAK242 (TLR4 antagonist) before LPS stimulation rescued such morphology alternations, suggesting that inflammatory milieu could promote platelet activation in a TLR4-dependent manner (Figure 11A). Next, NTA was conducted to measure the total concentration of platelet-derived EVs in the same volume of supernatant (1 ml). As shown in Figure 11B, LPS-stimulated platelets secreted more EVs compared to PBS control, which was suppressed by TAK242 treatment. More importantly, TAK242 also repressed LPS-activated TLR4/MyD88/ARF6 signal axis in the platelets (Figure 11C). Furthermore, EVs from TAK242-pretreated platelets before LPS stimulation (LPS+TAK242-EVs) reversed the phosphorylation of ERK, Smad3 and p53 in HK-2 cells (Figure 11D). According to previous studies, IκB kinase phosphorylation controls platelet secretion 27,28. We observed that IκB phosphorylation was dramatically inhibited by BMS and BAY (IKK inhibitors) associated with downregulated protein levels of SNARE complex of STX-2 and SNAP 23 in LPS-stimulated platelets (Figure 11E). To further analyze the effect of IKK inhibition in septic AKI *in vivo*, we administered BMS (10 mg/kg) to the mice 2 h before CLP. BMS treatment significantly reduced the levels of SCr and BUN (Figure 11H-I) and decreased the protein concentration of EVs in plasma (Figure 11J). Moreover, BMS significantly attenuated pathological damage in the kidneys after CLP (Figure 11F-G). Taken together, these findings demonstrated that sepsis activated platelets via TLR4, and that further platelet secretion was controlled by IKK activity, which could become a therapeutic target for managing septic AKI.

## Discussion

**Figure 12:**
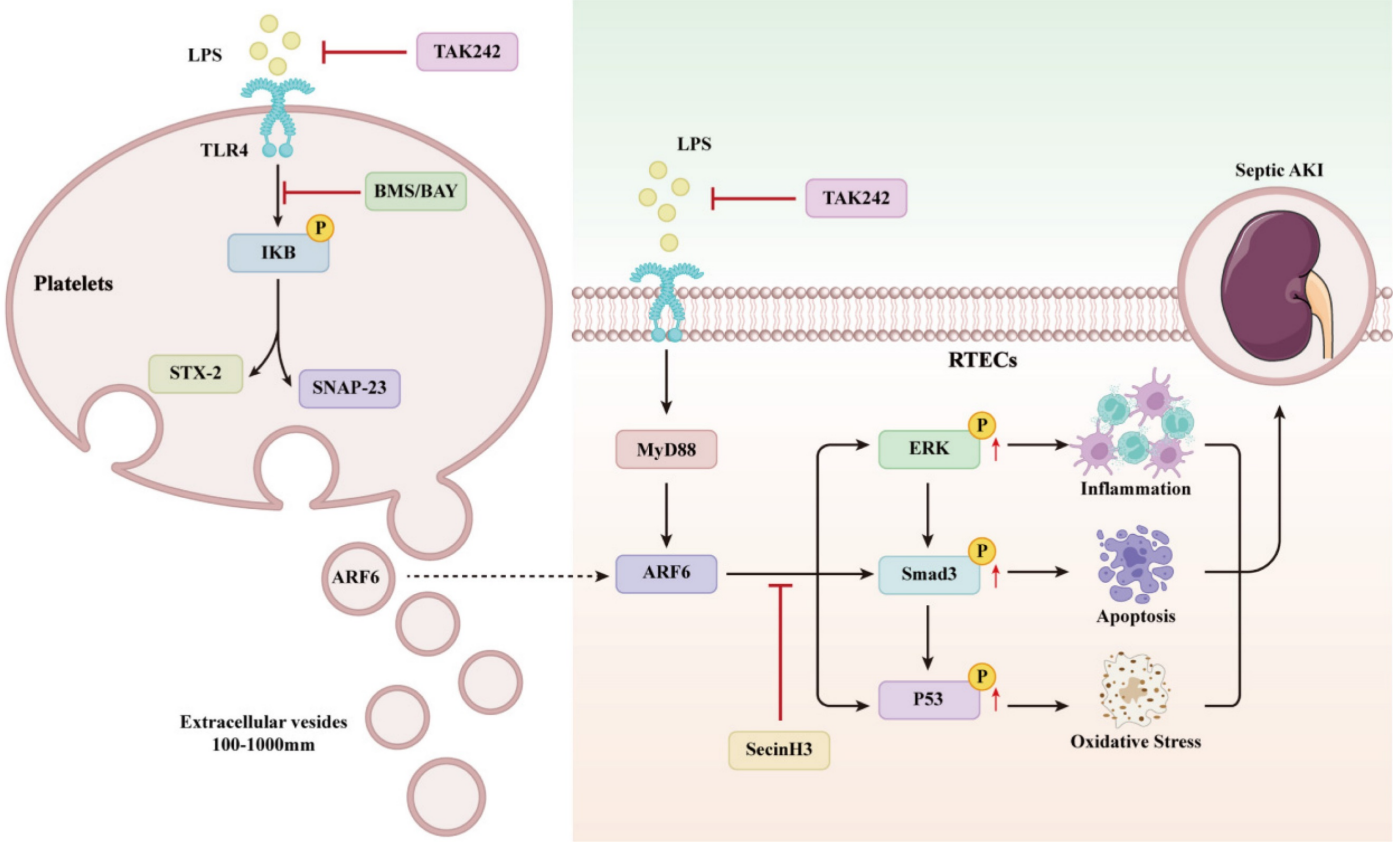
The mechanisms of platelet-derived EVs on septic AKI.

Platelet-derived EVs are considered to take part in the intercellular communication between platelets and recipient cells during sepsis 24-26,46,47. Here, we reported that purified EVs from LPS-activated platelets aggravate septic AKI via promoting inflammation, apoptosis, and oxidative stress (Figure 12).

Our results showed that sepsis significantly promoted platelet secretion, which was further confirmed *in vitro* as we observed dramatically increased levels of EVs by LPS, indicating that inflammatory milieu contributed to platelet activation and secretion in sepsis. Confocal microscope and* in vivo* biodistribution imaging showed absorbance of platelet-derived EVs in RTECs and kidneys, indicating that platelet-derived EVs participated in the development of kidney disease. Moreover, inhibition of platelet secretion by BMS significantly attenuated CLP-injured damage in kidneys. Overall, these results supported a strong link between platelet-derived EVs and septic AKI.

According to previous studies, platelet-derived EVs were reported to play important roles in the progression of sepsis. Azevedo et al. showed that platelet-derived EVs from septic patients contribute to myocardial dysfunction via increasing myocardial nitrate content 25. Gambim et al. revealed that platelet-derived EVs induce endothelial cell apoptosis 24. Jiao et al. demonstrated that platelet-derived exosomes aggravate acute lung injury during sepsis via regulating Akt/mTOR autophagy pathway 26. Considering inflammation and apoptosis play critical roles in septic AKI, we then investigated whether platelet-derived EVs modulated kidney damage via inflammation and apoptosis during sepsis. Our results demonstrated that purified EVs from LPS-activated platelets aggravated sepsis-induced renal dysfunction, as evidenced by increased levels of SCr, BUN, and tubular injury scores. Furthermore, platelet-derived EVs stimulation induced apoptosis, promoted release of pro-inflammatory cytokines, and increased ROS production in RTECs. These alterations of RTECs stimulated by LPS-EVs were consistent with the characteristics of septic AKI.

Platelet-derived EVs contain different substances, including a variety of cytokines and chemokines, cytoskeleton components, microRNAs and mitochondrial DNA 48-51. Previous study confirmed that platelet-derived EVs could shuttle active substances to target cells. For an instance, Zhang et al. reported that glomerular endothelial cells can uptake chemokine ligand 7 (CXCL7) in platelet microparticles, contributing to the development of diabetic nephropathy 22. Next, we performed quantitative proteomics analysis to identify proteins between PBS-EVs and LPS-EVs. ARF6 was found to be significantly enriched in LPS-EVs in comparison to PBS-EVs. ARF6 is a small molecule of GTPase, which is responsible for cell endocytosis and exocytosis, membrane transport and actin cytoskeleton remodeling. The activity of ARF6 is switched when countering inflammation environment 52,53. As recent studies have suggested that ARF6 modulated inflammation and oxidative stress via activating β-catenin, mTORC1, and MAPKs during immune processes 54-56. Notably, the role of TGF-β/Smads pathway in kidney injury has recently attracted increasing attention. Both canonical and noncanonical signaling proteins can mediate TGF-β pathway 57. ERK as an important component in MAPK pathway plays critical roles in regulating cell inflammation and apoptosis 58. Wang et al. reported that Smad3 activation increases AKI sensitivity by interacting with p53 and NOX4 44. Che et al. demonstrated that the activation of ARF6/ERK/NOX4 axis promotes podocytes injury 43. Here, we found that the expression of activated ERK, Smad3 and p53 increased significantly after LPS-EVs stimulation, and that upregulated levels of these proteins promoted inflammation, apoptosis, and oxidative stress in RTECs, indicating that platelet-derived EVs aggravated septic AKI. Moreover, we reported that ARF6 activated ERK/Smad3/p53 axis in RTECs. Mechanistically, LPS-induced upregulation of ARF6 in renal tubular was dependent on TLR4/MyD88 signaling activation. As a PRP, TLR4 is activated by LPS and regulates inflammatory process and cell death during sepsis. Knockdown of TLR4 effectively suppressed the LPS-elevated expression of ARF6 and then inhibition of TLR4 or ARF6 significantly reduced inflammation, apoptosis, and oxidative stress in RTECs after LPS stimulation. Meanwhile, pharmacologic inhibition of ARF6 by SecinH3 *in vivo* alleviated septic AKI through reducing inflammation, apoptosis, and oxidative stress in kidneys. These results supported that ARF6 was a therapeutic target in septic AKI.

Finally, we suggested that the activity of IKK controlled platelet secretion, which was in accordance with previous research in which IκB kinase was found to mediate platelet secretion via phosphorylation of SNAP-23 27. There is now increasing evidence about IKK inhibition could suppress multiple organ dysfunction during sepsis 26,59,60, but the precise relationship requires to be further confirmed. Instead, it is still controversial about whether the inhibition of platelets could prevent the progression of sepsis 61-63. We reported that pharmacologically inhibition of IKK with BMS or BAY reduced SNAREs complex formation in platelets, which exerted a renoprotective effect during sepsis.

In summary, increased platelet-derived EVs during sepsis aggravated septic AKI through the ARF6 release to promote inflammation, apoptosis, and oxidative stress. The potential underlying mechanism was the activation of ERK/Smad3/p53 pathway by ARF6. Our findings may provide a therapeutic target for treating septic AKI.

## Supplementary Material

Supplementary figures and tables.
