# Supplementary material for: Platelet-derived extracellular vesicles aggravate septic acute kidney injury via delivering ARF6
Source: Int J Biol Sci. 2023 Oct 2;19(16):5055–73. doi: 10.7150/ijbs.87165 (PMC10620832; doi:10.7150/ijbs.87165)
Supplement: Supplementary file 1 — Supplementary figures and tables. [file ijbsv19p5055s1.pdf]

Supplementary tables

Table S1. Demographic data of the included healthy volunteers.

Variable	Results
Total, n	21
Age, years, median (IQR)	32 (26-34)
Gender, n (%)	
Male	11 (52.4)
Female	10 (47.6)
Hemoglobin, g/L, mean ± SD	126.9 ± 14.4
Albumin, g/L, mean ± SD	39.4 ± 3.2
BMI, kg/m^2, mean ± SD	22.3 ± 2.2
Platelets, 10^9/L, mean ± SD	233.1 ± 30.3

IQR, interquartile range; SD, standard deviation

Table S2. Primer sequences used in real-time PCR analysis.

Gene	Forward Primer	Reverse Primer
M-GAPDH	GCATGGCCTTCCGTGTTC	GATGTCATCATACTTGGCAGGTTT
M-KIM1	ACATATCGTGGAATCACAACGAC	ACTGCTCTTCTGATAGGTGACA
M-NGAL	GCAGGTGGTACGTTGTGGG	CTCTTGTAGCTCATAGATGGTGC
M-Bcl-2	TGTGAGGACCCAATCTGGAAA	TTGCAATGAATCGGGAGTTG
M-Bax	GATCAGCTCGGGCACTTTAG	TTGCTGATGGCAACTTCAAC
M-IL-1β	TGCCACCTTTTGACAGTGATG	AAGGTCCACGGGAAAGACAC
M-IL-6	AAAGAGTTGTGCAATGGCAATTCT	AAGTGCATCATCGTTGTTCATACA
M-TNF-α	CATCTTCTCAAAATTCGAGTGACAA	TGGGAGTAGACAAGGTACAACCC
M-MCP-1	CTTCTGGGCCTGCTGTTCA	CCAGCCTACTCATTGGGATCA
M-PGC1-α	CACCAAACCCACAGAAAACAG	GGGTCAGAGGAAGAGATAAAGTTG
M-ATP5a-1	CATTGGTGATGGTATTGCGC	TCCCAAACACGACAACTCC
M-NDUFS8	GTTCATAGGGTCAGAGGTCAAG	TCCATTAAGATGTCCTGTGCG
M-TOM20	GCTAAGGAGAGAGCTGGGCTTT	TGGTCCACACCCTTCTCGTAGT
H-GAPDH	GGAAGCTTGTCATCAATGGAAATC	TGATGACCCTTTTGGCTCCC
H-TLR4	GTCAGTGTGATTGTGGTATCC	ACCCAGTCCTCATTCTGACTC
H-ARF6	GGGAAGGTGCTATCCAAAATCTT	CACATCCCATACGTTGAACTTGA

M, denotes mice and H, denotes human

Table S3. Primary antibodies used in Western blot analysis.

Name	Company	Catalog number
Anti-HSP70	Abcam, MA, USA	ab194360
Anti-CD63	Abcam, MA, USA	ab252919
Anti-TSG101	Abcam, MA, USA	ab225877
Anti-CD81	Abcam, MA, USA	ab109201
Anti-Calnexin	Abcam, MA, USA	ab92573
Anti-NGAL	Abcam, MA, USA	ab125075
Anti-Bcl-2	Cell Signaling Technology, MA, USA	4223
Anti-Bax	Proteintech Group, Wuhan, China	50599-2-Ig
Anti-Cleaved caspase 3	Cell Signaling Technology, MA, USA	9664
Anti-Caspase 3	Cell Signaling Technology, MA, USA	9662
Anti-GAPDH	Abcam, MA, USA	ab128915
Anti-IL-1β	Santa Cruz Biotechnology, Texas, USA	sc-12742
Anti-TNF-α	Santa Cruz Biotechnology, Texas, USA	sc-12744
Anti-MCP-1	Santa Cruz Biotechnology, Texas, USA	sc-52701
Anti-IL-6	Proteintech Group, Wuhan, China	21865-1-AP
Anti-NOX4	Santa Cruz Biotechnology, Texas, USA	sc-518092
Anti-Nrf2	Santa Cruz Biotechnology, Texas, USA	sc-365949
Anti-ARF6	Proteintech Group, Wuhan, China	20225-1-AP
Anti-phospho-ERK	Cell Signaling Technology, MA, USA	4695
Anti-ERK	Cell Signaling Technology, MA, USA	4370
Anti-phospho-Smad3	Beyotime, Shanghai, China	AF1759
Anti-Smad3	Beyotime, Shanghai, China	AF1501
Anti-phospho-p53	Beyotime, Shanghai, China	AF5896
Anti-p53	Proteintech Group, Wuhan, China	10442-1-AP
Anti-TLR4	Servicebio, Wuhan, China	GB11519
Anti-MyD88	Servicebio, Wuhan, China	GB111554
Anti- β-actin	Proteintech Group, Wuhan, China	81115-1-RR
Anti-phospho-IκB	Santa Cruz Biotechnology, Texas, USA	sc-8404
Anti-IκB	Santa Cruz Biotechnology, Texas, USA	sc-1643
Anti-syntaxin 2	Santa Cruz Biotechnology, Texas, USA	sc-514642
Anti-SNAP 23	Santa Cruz Biotechnology, Texas, USA	sc-374215

Table S4. The sequences of siRNAs used in cell transfection.

Gene	Sense	Antisense
siTLR4	GCCGAAAGGUGAUUGUUGUTT	ACAACAAUCACCUUUCGGCTT
siARF6	GACGCCAUAAUCCUCAUCUTT	AGAUGAGGAUUAUGGCGUCTT
siNC	UUCUCCGAACGUGUCACGUTT	ACGUGACACGUUCGGAGAATT

Figure S1. Effect of different doses of LPS on HK-2 cells. HK-2 cells were treated with LPS (0-100 μg/ml) for 24 h. (A) The cell viability of HK-2 cells treated with different concentrations of LPS (0, 5, 10, 20, 50, and 100 μg/ml) for 24 h (n = 6). (B) Representative flow cytometric plots of HK-2 cells. (C) Quantitative analysis of apoptosis rate of HK-2 cells (n = 3). *P < 0.05, **P < 0.01, ***P < 0.001.

Figure S2. Effect of platelet-derived EVs on HK-2 cells apoptosis. The apoptosis of HK-2 cells treated with PBS, LPS, LPS+PBS-EVs, LPS+LPS-EVs, and LPS+LPS-EVs-free supernatant was determined by flow cytometry (n = 3). ***P < 0.001 ; $^{\#\#}$P < 0.01.

Figure S3. Impact of PBS-EVs and LPS-EVs on kidneys and HK-2 cells. (A-B) Representative PAS staining images (scale bar, 50 μm) and tubular injury scores of Control, PBS-EVs and LPS-EVs (n = 5). (C-D) SCr and BUN levels in different gruops (n = 5). (E) Quantitative real-time PCR analysis of the mRNA expression of KIM1 and NGAL in kidneys (n = 3). (F) Western blotting and densitometry analysis of NGAL in kidneys (n = 3). (G) The cytotoxic effect of PBS-EVs or LPS-EVs on HK-2 cells was determined by CCK8 assay (n = 6). (H-I) Representative flow cytometric plots and quantitative analysis of apoptosis rate of HK-2 cells (n = 3). (J) Western blotting and densitometry analysis of Bcl-2, Bax, Cas 3, and C-Cas 3 in HK-2 cells (n = 3). (K-M) The concentrations of TNF-α, IL-6, and IL-1β in the supernatant of HK-2 cells (n = 6). ***P < 0.001.

Figure S4. Isolation and characterization of plasma EVs in mice CLP was conducted in mice and blood samples were collected for isolation of plasma EVs by using exosome precipitation solution according to the manufacturer's protocols. (A) Images of Sham-EVs and CLP-EVs detected by TEM (scale bar, 100 nm). (B-C) The size distribution and particle concentration of Sham-EVs and CLP-EVs analyzed by NTA (n = 3). (D) The protein concentration of Sham-EVs and CLP-EVs derived from 1 ml of plasma was determined by BCA assay (n = 3). (E-F) Western blotting and densitometry analysis of protein levels of HSP70, CD63, TSG101, CD81 in Sham-EVs and CLP-EVs (n = 3). (G-H) Representative flow cytometric plots and quantitative analysis of CD61 positive EVs (n = 3). **P < 0.01, ***P < 0.001.

Figure S5. CLP-EVs impaired renal function via promoting apoptosis and inflammation in mice. Plasma EVs (100 μg) isolated from Sham and CLP mice were transferred into WT mice and blood samples and kidneys were collected for further analysis 4 h later. (A) The animal protocol schematic for infusion of Sham-EVs or CLP-EVs to WT mice. (B-C) SCr and BUN levels in different groups (n = 5). (D-E) Representative PAS staining images (scale bar, 50 μm) and tubular injury scores of different groups (n = 5). (F) Quantitative real-time PCR analysis of the mRNA expression of KIM1 and NGAL in kidneys (n = 3). (G) Western blotting and densitometry analysis of NGAL in kidneys (n = 3). (H) Representative TUNEL and Ly6G staining images in kidneys (scale bar, 50 μm). (I) Quantitative analysis of TUNEL positive cells in kidneys (n = 5). (J) Quantitative analysis of Ly6G positive cells in kidneys (n = 5). (K) The mRNA expression of Bcl-2, Bax and Bcl-2/Bax in kidneys analyzed by quantitative real-time PCR (n = 3). (L) Western blotting and quantification by densitometry of Bcl-2, Bax, Cas 3, and C-Cas 3 in kidneys (n = 3).

(M) The mRNA expression of IL-6, MCP-1, TNF-α and MCP-1 in kidneys analyzed by quantitative real-time PCR (n = 3). (N) Western blotting and densitometry analysis of the protein levels of IL-6, MCP-1, TNF-α and MCP-1 in kidneys (n = 3). ***P < 0.001.

Figure S6. Effect of plasma EVs on TCMK-1 cells. Isolated plasma EVs from Sham or CLP mice were subjected to TCMK-1 cells and were co-cultured for 4 h. (A) The protocol schematic for administration of Sham-EVs or CLP-EVs to TCMK-1 cells. (B) The cell viability of TCMK-1 cells treated with Sham-EVs or CLP-EVs for 4 h (n = 6). (C-D) Representative flow cytometric plots and quantitative analysis of apoptosis rate of TCMK-1 cells (n = 3). (E) Western blotting and quantification by densitometry of Bcl-2, Bax, Cas 3, and C-Cas 3 in kidneys (n = 3). (F) The concentrations of TNF-α, IL-6, and IL-1β in the supernatant of TCMK-1 cells (n = 6). **P < 0.01, ***P < 0.001.

Figure S1

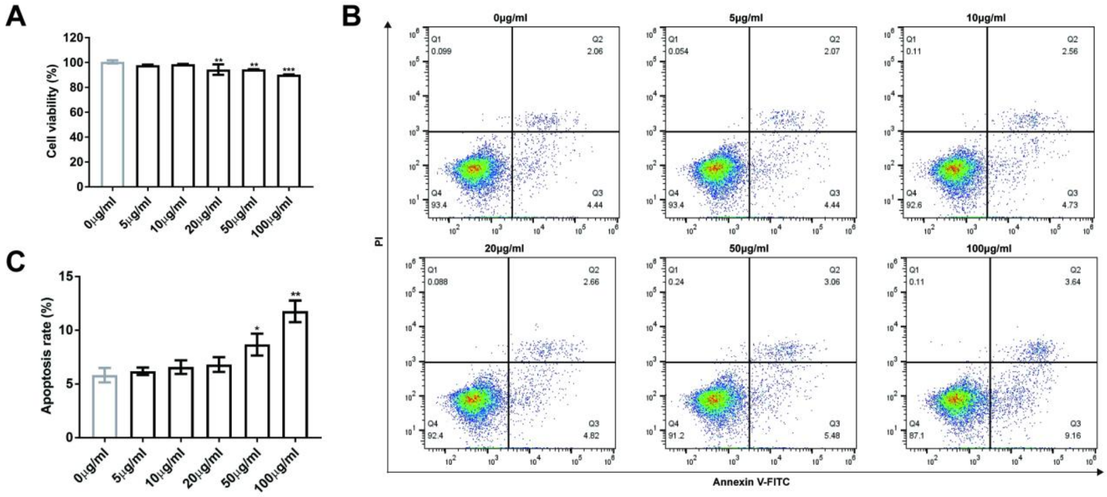

Figure S2

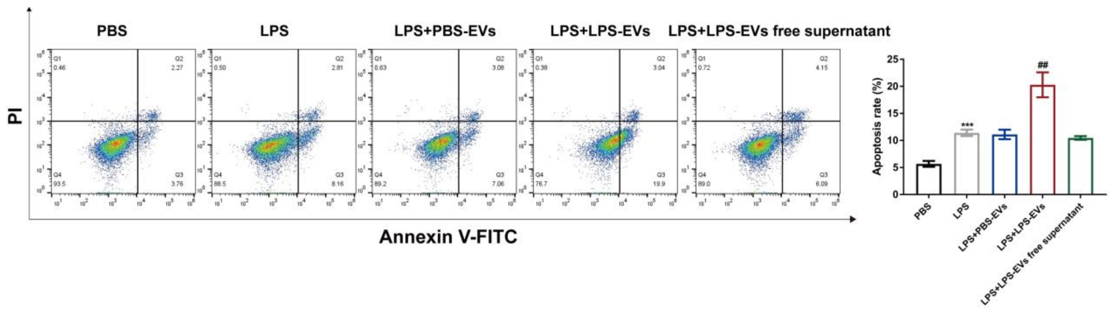

Figure S3

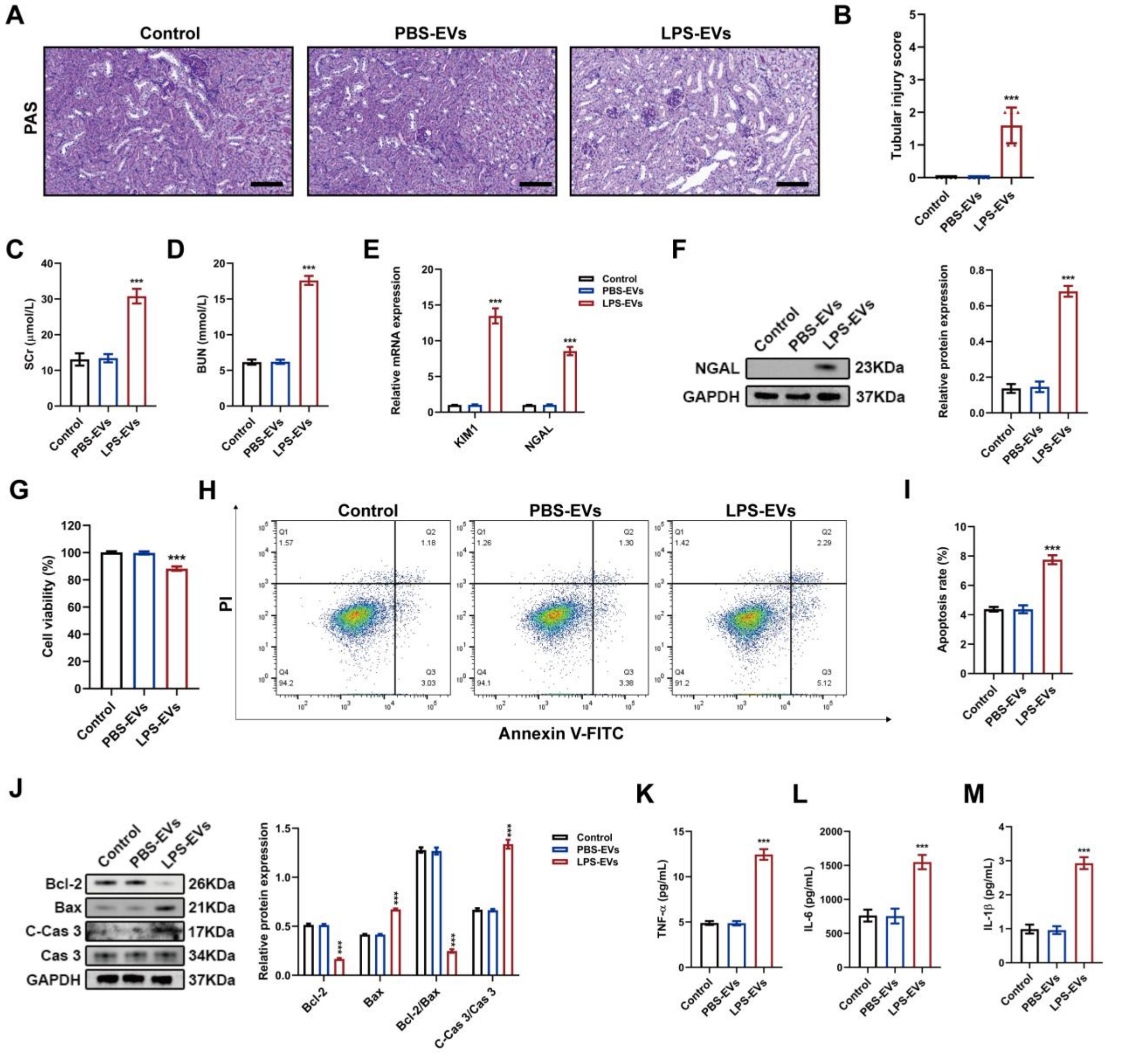

A
Control
PBS-EVs
LPS-EVs
PAS
B
Tubular injury score
C
SCr (μmol/L)
D
BUN (mmol/L)
E
Relative mRNA expression
KIM1
NGAL
F
NGAL 23KDa
GAPDH 37KDa
Relative protein expression
G
Cell viability (%)
H
PI
Annexin V-FITC
I
Apoptosis rate (%)
J
Bcl-2 26KDa
Bax 21KDa
C-Cas 3 17KDa
Cas 3 34KDa
GAPDH 37KDa
Bcl-2/Bax
C-Cas 3/Cas 3
K
TNF-α (pg/mL)
L
IL-6 (pg/mL)
M
IL-1β (pg/mL)

Figure S4

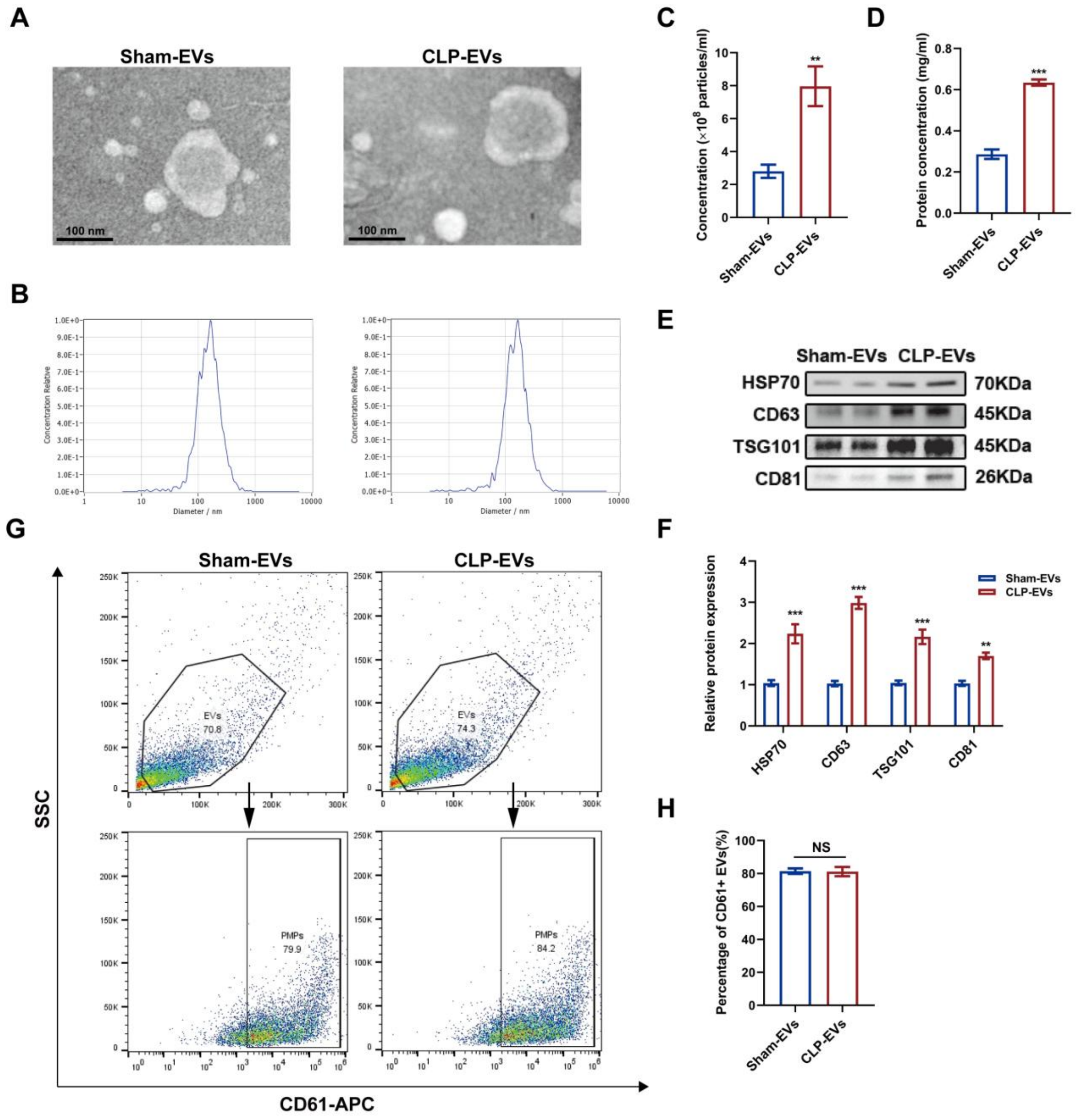
A
Sham-EVs
CLP-EVs
100 nm
100 nm
B
Concentration Relative
Diameter / nm
C
Concentration (×10^8 particles/ml)
Sham-EVs
CLP-EVs
**
D
Protein concentration (mg/ml)
Sham-EVs
CLP-EVs
***
E
Sham-EVs CLP-EVs
HSP70 70KDa
CD63 45KDa
TSG101 45KDa
CD81 26KDa
F
Relative protein expression
Sham-EVs
CLP-EVs
HSP70
CD63
TSG101
CD81
G
Sham-EVs
CLP-EVs
EVs
70.8
EVs
74.3
PMPs
79.9
PMPs
84.2
SSC
CD61-APC
H
Percentage of CD61+ EVs(%)
NS
Sham-EVs
CLP-EVs

Figure S5

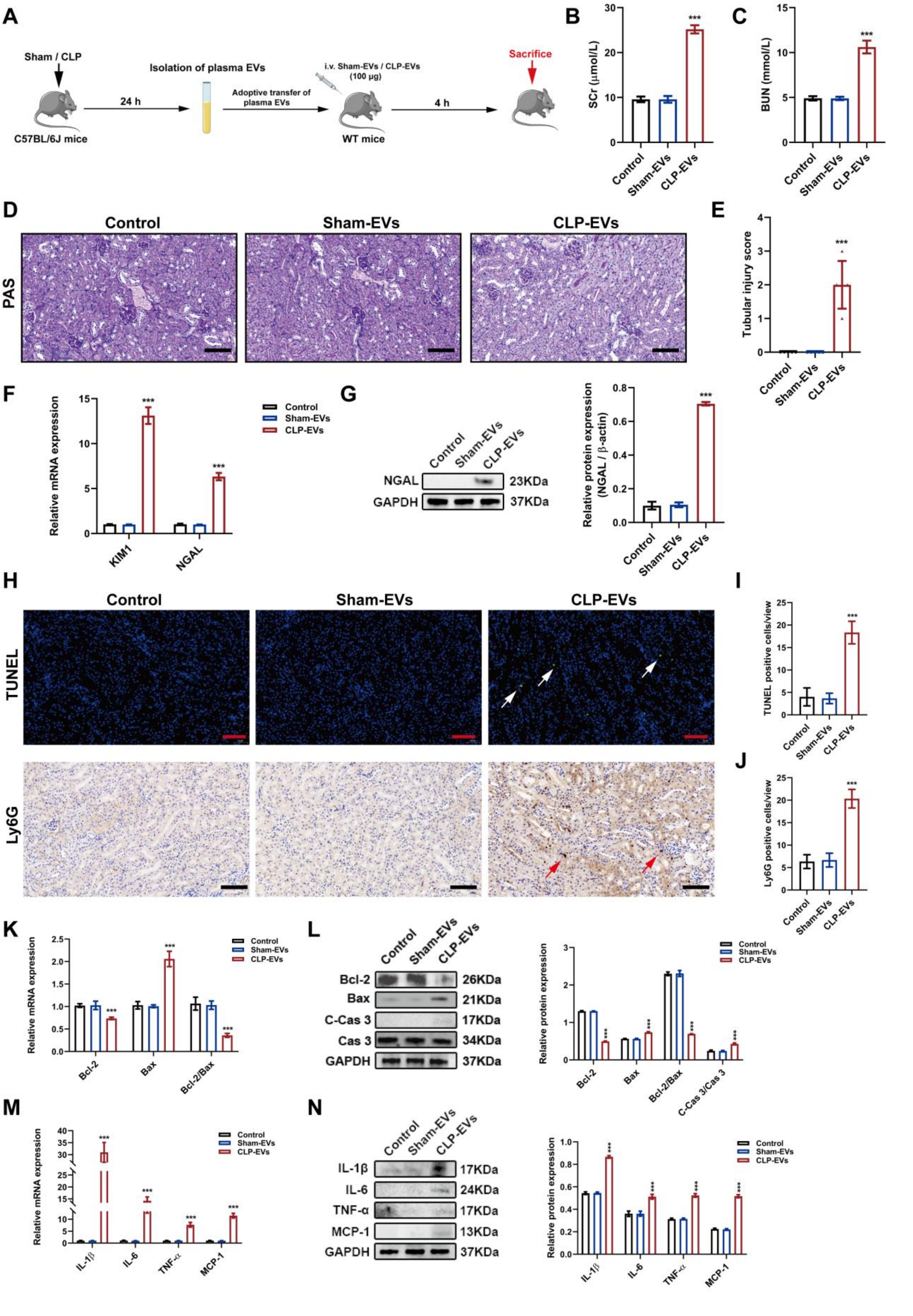
A
Sham / CLP
C57BL/6J mice
24 h
Isolation of plasma EVs
Adoptive transfer of plasma EVs
i.v. Sham-EVs / CLP-EVs (100 μg)
WT mice
4 h
Sacrifice
B
SCr (μmol/L)
C
BUN (mmol/L)
Control
Sham-EVs
CLP-EVs
D
PAS
E
Tubular injury score
F
Relative mRNA expression
KIM1
NGAL
G
NGAL 23KDa
GAPDH 37KDa
Relative protein expression (NGAL / β-actin)
H
TUNEL
Ly6G
I
TUNEL positive cells/view
J
Ly6G positive cells/view
K
Bcl-2
Bax
Bcl-2/Bax
L
Bcl-2 26KDa
Bax 21KDa
C-Cas 3 17KDa
Cas 3 34KDa
GAPDH 37KDa
Relative protein expression
C-Cas 3/Cas 3
M
IL-1β
IL-6
TNF-α
MCP-1
N
IL-1β 17KDa
IL-6 24KDa
TNF-α 17KDa
MCP-1 13KDa
GAPDH 37KDa
***

Figure S6

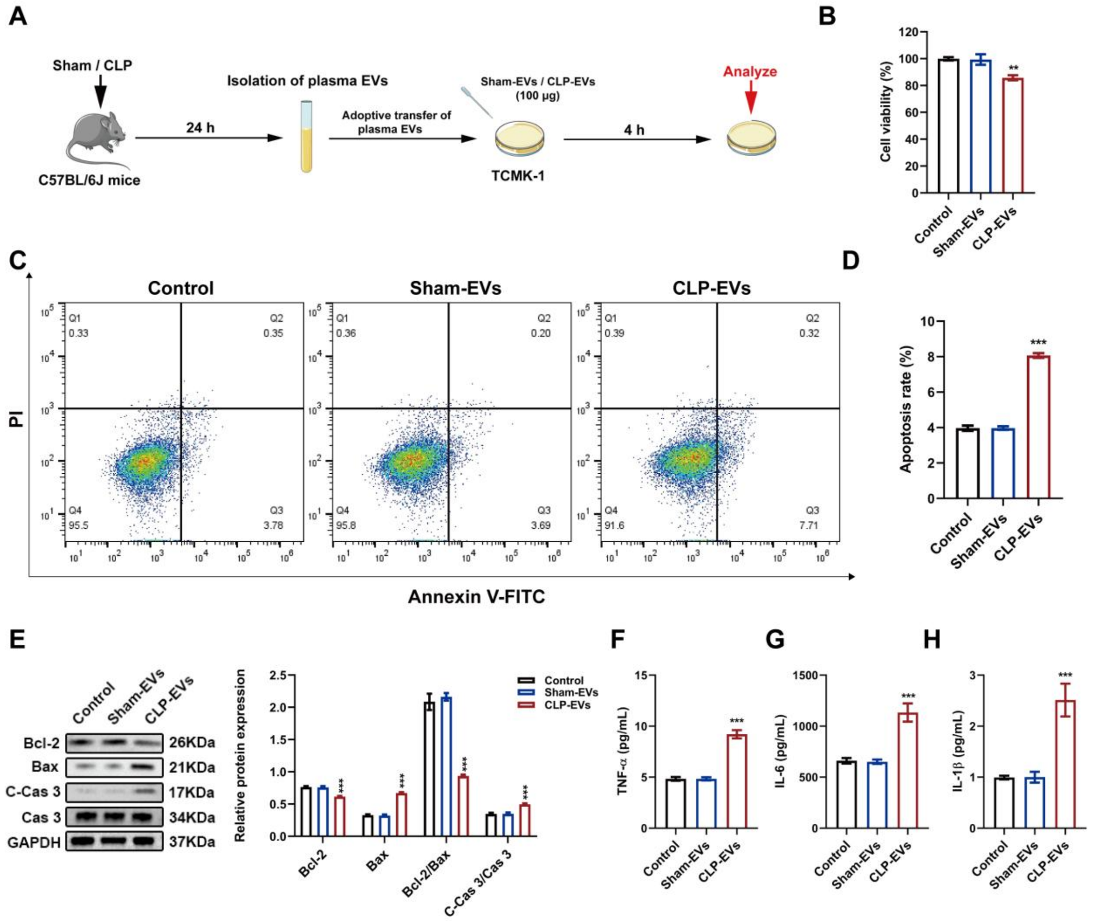
A
Sham / CLP
C57BL/6J mice
24 h
Isolation of plasma EVs
Adoptive transfer of plasma EVs
Sham-EVs / CLP-EVs (100 μg)
TCMK-1
4 h
Analyze
B
Cell viability (%)
Control
Sham-EVs
CLP-EVs
C
Control
Sham-EVs
CLP-EVs
PI
Annexin V-FITC
Q1 0.33
Q2 0.35
Q3 3.78
Q4 95.5
Q1 0.36
Q2 0.20
Q3 3.69
Q4 95.8
Q1 0.39
Q2 0.32
Q3 7.71
Q4 91.6
D
Apoptosis rate (%)
E
Bcl-2 26KDa
Bax 21KDa
C-Cas 3 17KDa
Cas 3 34KDa
GAPDH 37KDa
Relative protein expression
Bcl-2
Bax
Bcl-2/Bax
C-Cas 3/Cas 3
F
TNF-α (pg/mL)
G
IL-6 (pg/mL)
H
IL-1β (pg/mL)
